# In Vitro and In Vivo Antiviral Studies of New Heteroannulated 1,2,3-Triazole Glycosides Targeting the Neuraminidase of Influenza A Viruses

**DOI:** 10.3390/ph15030351

**Published:** 2022-03-14

**Authors:** Omnia Kutkat, Ahmed Kandeil, Yassmin Moatasim, Yaseen A. M. M. Elshaier, Wael A. El-Sayed, Samir T. Gaballah, Ahmed El Taweel, Mina Nabil Kamel, Mohamed El Sayes, Mohammed A. Ramadan, Rabeh El-Shesheny, Farouk M. E. Abdel-Megeid, Richard Webby, Ghazi Kayali, Mohamed A. Ali

**Affiliations:** 1 Center of Scientific Excellence for Influenza Viruses, National Research Centre, Giza 12622, Egypt; omnia.abdelaziz@human-link.org (O.K.); yasmin.moatasim@human-link.org (Y.M.); ahmed.nageh@human-link.org (A.E.T.); mina@human-link.org (M.N.K.); mohameddiaaelsayes@outlook.com (M.E.S.); rabeh.elshesheny@human-link.org (R.E.-S.); 2 Department of Infectious Diseases, St. Jude Children’s Research Hospital, Memphis, TN 38105, USA; richard.webby@stjude.org; 3 Department of Organic and Medicinal Chemistry, Faculty of Pharmacy, University of Sadat City, Menoufia 32897, Egypt; yaseen.elshaier@fop.usc.edu.eg; 4 Photochemistry Department, National Research Centre, Giza 12622, Egypt; waelshendy@gmail.com (W.A.E.-S.); samir.gaballah@gmail.com (S.T.G.); faroukezat@yahoo.com (F.M.E.A.-M.); 5 Department of Chemistry, College of Science, Qassim University, Buraydah 52571, Saudi Arabia; 6 Department of Microbiology and Immunology, Faculty of Pharmacy, Cairo University, Cairo 12613, Egypt; m_ramadan56@hotmail.com; 7 Department of Life Sciences, Human Link, Dubai 48800, United Arab Emirates

**Keywords:** glycosides, heterocyclic, human H1N1, antiviral, avian H5N1, neuraminidase inhibitor

## Abstract

There is an urgent need to develop and synthesize new anti-influenza drugs with activity against different strains, resistance to mutations, and suitability for various populations. Herein, we tested in vitro and in vivo the antiviral activity of new 1,2,3-triazole glycosides incorporating benzimidazole, benzooxazole, or benzotriazole cores synthesized by using a click approach. The Cu-catalyzation strategy consisted of 1,3-dipolar cycloaddition of the azidoalkyl derivative of the respective heterocyclic and different glycosyl acetylenes with five or six carbon sugar moieties. The antiviral activity of the synthesized glycosides against wild-type and neuraminidase inhibitor resistant strains of the avian influenza H5N1 and human influenza H1N1 viruses was high in vitro and in mice. Structure–activity relationship studies showed that varying the glycosyl moiety in the synthesized glycosides enhanced antiviral activity. The compound (2*R*,3*R*,4*S*,5*R*)-2-((1-(Benzo[d]thiazol-2-ylmethyl)-1*H*-1,2,3-triazol-4-yl)methoxy)tetrahydro-2*H*-pyran-3,4,5-triyl triacetate (Compound **9c**) had a 50% inhibitory concentration (IC_50_) = 2.280 µM and a ligand lipophilic efficiency (LLE) of 6.84. The compound (2*R*,3*R*,4*S*,5*R*)-2-((1-((1*H*-Benzo[d]imidazol-2-yl)methyl)-1*H*-1,2,3-triazol-4-yl)methoxy)tetrahydro-2*H*-pyran-3,4,5-triyl triacetate had IC_50_ = 2.75 µM and LLE = 7.3 after docking analysis with the H5N1 virus neuraminidase. Compound **9c** achieved full protection from H1N1 infection and 80% protection from H5N1 in addition to a high binding energy with neuraminidase and was safe in vitro and in vivo. This compound is suitable for further clinical studies as a new neuraminidase inhibitor.

## 1. Introduction

Influenza A viruses (IAVs), including the H1N1 and H3N2 subtypes, are the major cause of seasonal influenza epidemics and occasional pandemics in humans [1]. Several subtypes of avian influenza viruses (AIVs) have emerged during the last two decades and caused human infections. The two main control strategies against IAV are vaccination and specific antiviral drugs [2]. Currently, several FDA-approved antiviral drugs are commercially available to treat IAV, including neuraminidase inhibitors (NAIs) (zanamivir, peramivir, and oseltamivir phosphate) [3], M2 ion channel blockers (amantadine and rimantadine) [4], and RNA polymerase inhibitors (favipiravir and Xofluza) [5]. The continuous evolution of IAV and the overuse of antiviral drugs have led to the emergence of variant influenza strains that are resistant to amantadine, rimantadine, oseltamivir, and zanamivir, raising public health concerns and highlighting an urgent need to develop new anti-influenza drugs [6].

The neuraminidase glycoprotein (NA) remains the most attractive target because it is a surface glycoprotein, making it more accessible to anti-influenza drugs. Numerous sialic (neuraminic) acid analogs that competitively bind to the NA active site and potently inhibit enzyme activity have been synthesized [7] and have been found to stop the activity of the receptor-cleaving enzyme (sialidase), which binds to and cleaves the terminal neuraminic (sialic) acid moiety from oligosaccharide chains [8]. The design of NAIs through docking is based on the use of the conserved structure of the NA active site and on the compound fitting inside the binding site of the protein’s molecular surface [9].

Finding novel, potent leads in viral therapy has become the main objective in the development of antivirals in the last decades. The click synthesis strategy is considered to be one of the most efficient approaches for synthesizing new targeted bioorganic compounds for use as antiviral agents [10]. Following the strategy of the cycloaddition reaction catalyzed by Cu (I) ions in which a functionalized azide reacts with an alkyne derivative under mild conditions, the targeted 1,4-disubstitued 1,2,3-triazole with the desired functionalities were selectively afforded in satisfactory yields [11]. The selective formation of functionalized 1,2,3-triazole core is an interesting feature as this motif exists in various compounds reported to possess anti-HIV [12] or anti-bacterial activity [13,14]. Furthermore, triazole-modified analogs possessing significant antiviral action, such as zanamivir, have been prepared by applying the catalyzed azide–alkyne click reaction [15]. The high dipole moment and the ability of H-bonding formation in addition to the plain stability of the triazole ring to metabolic variation enhance its potential efficiency as a connecting group [16,17]. Nucleosides and structural analogs including glycosyl heterocycles have been shown to have antiviral activity [18,19,20,21,22,23,24,25,26,27] by inhibiting the involved enzymes, and several of them performed their catalytic activity with low cytotoxicity and a high selectivity index.

**Figure 1:**
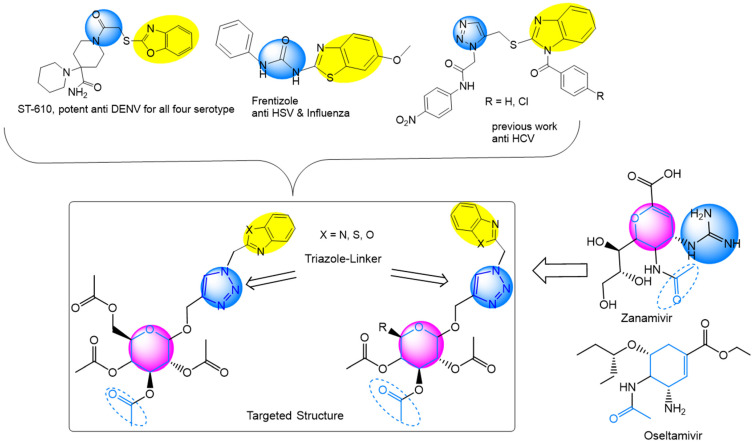
Medicinal chemistry rational and structures of zanamivir, oseltamivir, and selected antiviral candidates.

Compounds incorporating small heterocyclic ring systems, such as imidazole, oxazole, and thiazole, have gained considerable interest in antiviral drug discovery research. Studies have revealed that benzoxazole targeted NS3 helicase and inhibited dengue virus replication by targeting the capsid protein [28,29]. Benzothiazole plays a vital role in the design and development of antiviral drugs [30]. Benzimidazole derivatives have various biological activities that may inhibit a wide variety of viruses [31]. This work strategy is based on the nucleoside formation of three forms of carbohydrates with heteroannulated systems linked by a triazole moiety (Figure 1).

The co-crystallized ligand zanamivir (Relenza) has been used because it shows less resistance and has fewer side effects than adamantane groups [6,32]. All H3N2 viruses show adamantine resistance but are still sensitive to neuraminidase inhibitors; seasonal H1N1 virus resistance to oseltamivir was nearly 100% during 2009–2010 [8]. This highlights the urgent need to develop new anti-influenza drugs, particularly with the emergence of variants in the amino acid composition of different proteins of influenza virus causing multidrug resistance in humans.

In this study, zanamivir was used as a co-crystal in the docking study and in the synthesis of analogs for compounds used as anti-influenza A through the inhibition of neuraminidase enzyme activity. The aim of the current study was to investigate the antiviral activity of hybrid molecules formed via the synthesis of a series of glycosyl-1,2,3-triazoles linked to other heterocyclic cores and sugar moieties. Varying the nature of the heterocyclic motif and the carbohydrate moiety was useful as a way of correlating the activity results with the characteristic structural features of the synthesized derivatives. Newly synthesized compounds were tested in vitro and in vivo against AI H5N1 wild-type and resistant strains carrying mutations that cause resistance to NAIs as well as the human H1N1 virus. Here, we summarize the chemoinformatics and chemistry data for the design and synthesis of various new anti-influenza drugs to avoid resistance and side effects.

## 2. Results

### 2.1. Synthesis of the Designed Compounds

**Figure 2:**
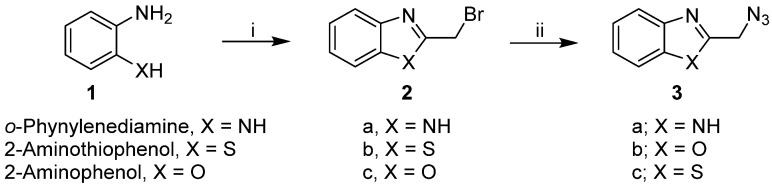
Preparation of 2-(azidomethyl)benzo[d]imidazole, 2-(azidomethyl)benzo[d]thiazole, and 2-(azidomethyl)benzo[d]oxazole. Synthetic procedures: (i) X = NH: bromoacetic acid, 4N HCl, reflux, 4 h, NH_4_OH; X = S or O: chloroacetyl chloride or bromoacetyl bromide, trimethylamine, xylene, 0 °C for 2 h, reflux for 8 h. (ii) ethanol/DMSO/water (8:1:1), NaN_3_, 65–75 °C, 7 h.

**Figure 3:**
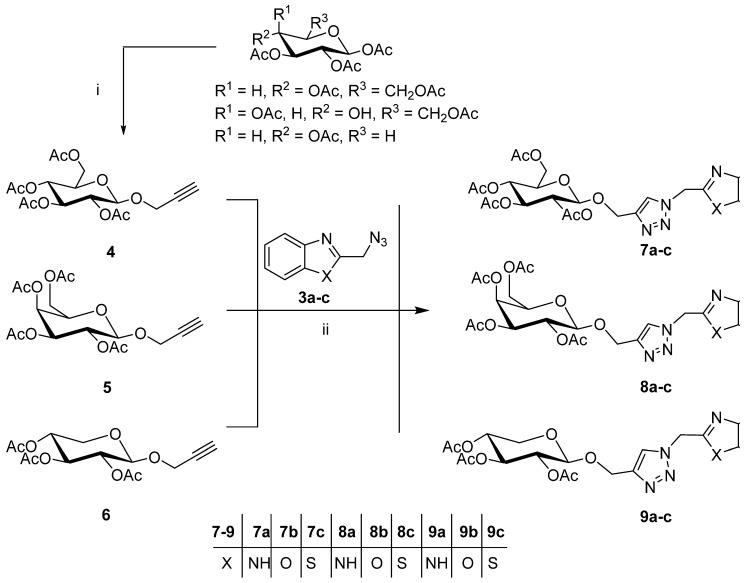
Synthetic procedure: (i) Propargyl alcohol, BF3.Et2O, CH2Cl2, rt., (ii) CuL3Br, 2-butanol, rt, 48 h.

We synthesized a series of substituted triazole glycosides with different carbohydrate moieties linked to benzimidazole, benzoxazole, or benzotriazole ring systems with 1,2,3-triazole linkers. The 2-bromomethyl-derivatives of benzimidazole (**2a**), benzoxazole (**2b**), and benzothiazole (**2c**) were prepared via an adapted procedure as shown in Figure 2 [33,34,35]. Azidation of the heteroaryl bromide **2a**–**c** was accomplished in hot ethanol/DMF/water in quantitative yields, as previously reported [36,37] (Figure 2). The afforded azidomethyl derivatives of benzimidazole, benzoxazole, and benzothiazole, **3a**–**c** were then allowed to react with terminal acetylenic glucopyranoside sugars **4**–**6**, incorporating the acetylated d-glucose, d-galactose, or d-xylose moiety, under modified copper-catalyzed click 1,3-dipolar cycloaddition to produce the corresponding 1,2,3-triazole-N-linked glycosides **7**–**9**, in 84–96% yields (Figure 3).

### 2.2. Antiviral Efficacy of Synthetic Compounds

#### 2.2.1. Cytotoxicity of Synthetic Compounds, Oseltamivir, and Zanamivir

**Figure 4:**
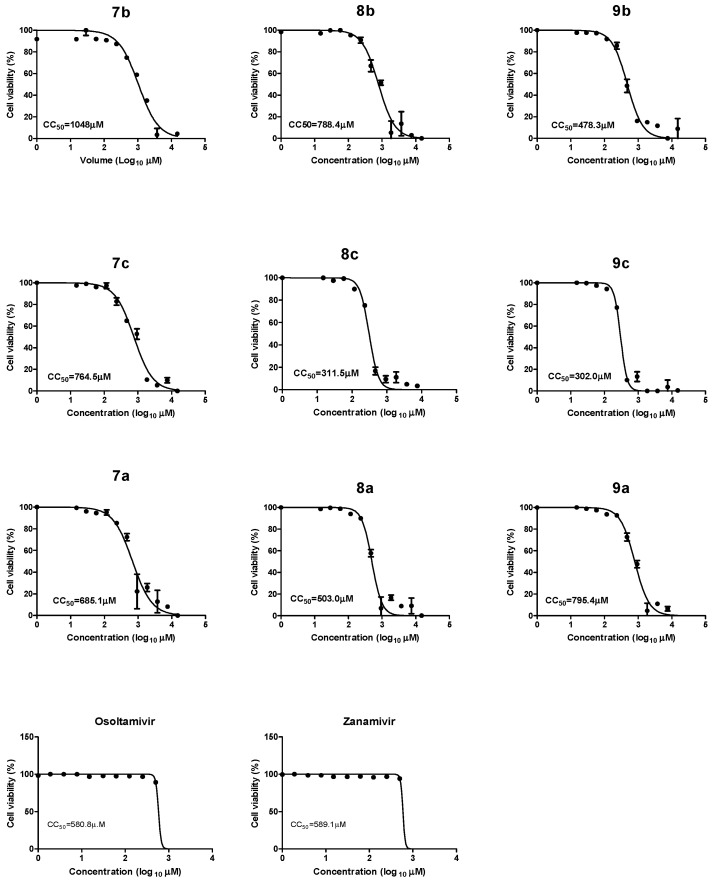
Cytotoxicity assay of new synthetic compounds, oseltamivir, and zanamivir in MDCK cells. The 50% cytotoxic concentration (CC_50_) of each tested compound was calculated by using nonlinear regression analysis (GraphPad Prism software).

The cytotoxicity of the synthesized compounds, oseltamivir, and zanamivir was evaluated in MDCK cells via (3-[4,5-dimethylthiazol-2-yl]-2,5 diphenyl tetrazolium bromide) (MTT) assay. The compounds were essentially non-toxic for MDCK cells up to a dose of 1048 µM for **7b** but only up to 302 µM for **9c**, compared to 580.8 µM and 589.1 µM for oseltamivir and zanamivir, respectively (Figure 4). The toxic effects of the tested compounds were dose-dependent, as shown by the 50% cytotoxic concentrations (CC_50_) (Table 1). We selected the safe concentrations for use in the subsequent antiviral studies.

#### 2.2.2. Antiviral Properties

**Figure 5:**
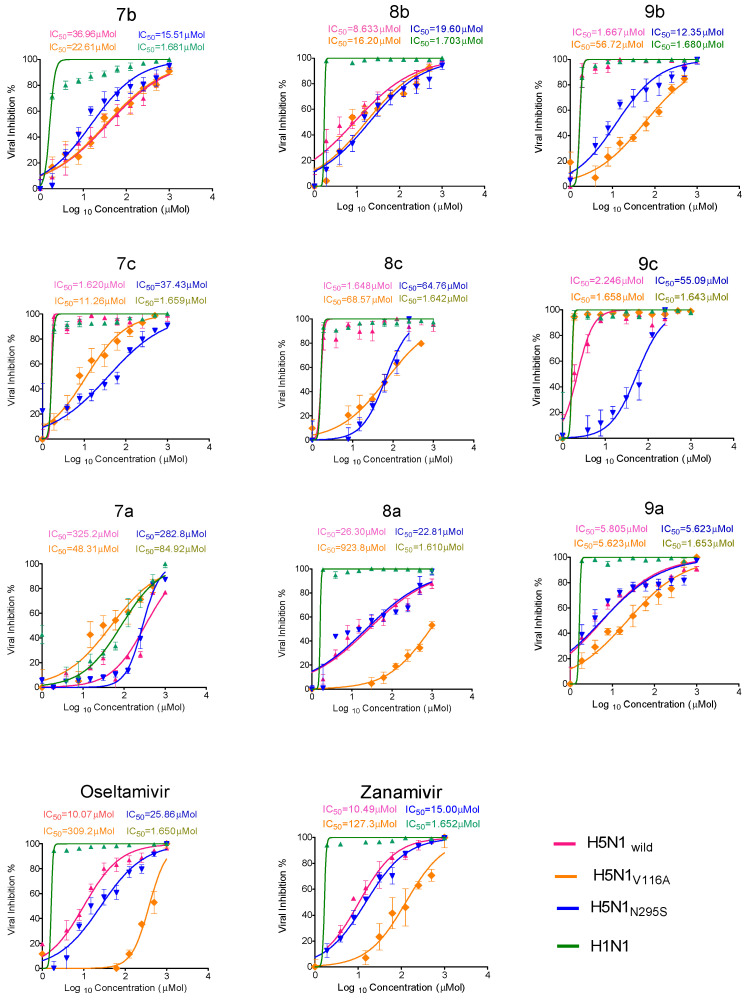
The 50% Inhibitory concentration (IC_50_) of new synthetic compounds, oseltamivir, and zanamivir against H5N1_wild_, H5N1_V116A_, H5N1_N295S_, and H1N1 viruses.

The antiviral properties of the synthesized compounds were tested against highly pathogenic A/chicken/Egypt/B13825A/2017 (H5N1_wild_), H5N1_V116A_, H5N1_N295S_, and H1N1 viruses using the crystal violet assay as described in the methodology section [38,39] and were then compared with oseltamivir and zanamivir as the standard for NAIs. We calculated the 50% inhibitory concentration (IC_50_) value for each synthesized and control compound against four tested viruses (Figure 5). The promising compounds had antiviral activity against H5N1_wild_, H5N1_V116A_, and H5N1_N295S_, with IC_50_ levels lower than those of oseltamivir and zanamivir against the H1N1 virus. All tested compounds had antiviral activity except **7a**. The selectivity index (SI) is the ratio of CC_50_ to IC_50_ (Table 1) and is an important parameter identifying the selectivity of the tested compounds for the tested virus rather than the normal host cell. From the observed results, it can be concluded that many of the newly synthesized compounds had antiviral activity with a potency higher than that of oseltamivir and zanamivir, the standard NAIs. Compounds **9a**, **8b**, and **9c** were superior among the tested compounds.

The developed synthetic compounds had a high selectivity index (SI ≥ 100) with one or more than one virus, as the **9c** compound had an SI of 134.4, 183, 5.4, and 184 when it was tested against the H5N1_wild_, H5N1_V116A_, H5N1_N295S_, and H1N1 viruses, respectively. Moreover, the **9a** compound had an SI of 137 to 482 when it was tested against the viruses. Compound **8b** had SI = 91.3, 48.6, 40.2, and 463.7 when it was tested against the H5N1_wild_, H5N1_V116A_, H5N1_N295S_, and H1N1, viruses, respectively. The SI for oseltamivir was 57.5, 1.8, 22.5, and 352 against the H5N1_wild_, H5N1_V116A_, H5N1_N295S_ and H1N1 viruses, respectively and 56.6, 4.6, 39.2, and 356.56 against the H5N1_wild_, H5N1_V116A_, H5N1_N295S_ and H1N1 viruses, respectively, when zanamivir was tested.

#### 2.2.3. Antiviral Properties According to the Plaque Reduction Assay

**Figure 6:**
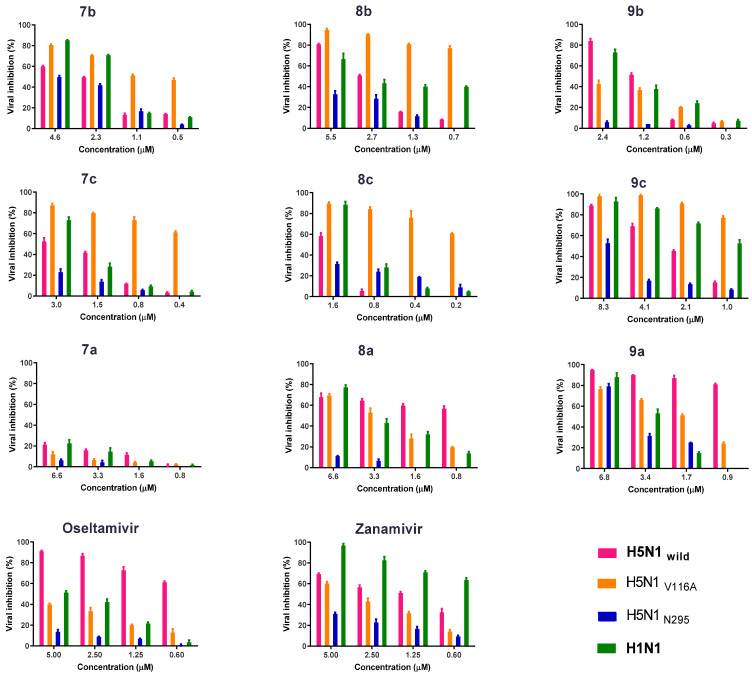
Antiviral activity of the new synthetic compounds, oseltamivir, and zanamivir against the H5N1_wild_, H5N1_V116A_, H5N1_N295S_, and H1N1 viruses using a plaque reduction assay.

The antiviral activities of the synthesized compounds and NAIs (oseltamivir and zanamivir) against the H5N1_wild_, H5N1_V116A_, H5N1_N295S_, and H1N1 viruses were determined via plaque reduction assay. The NAIs’ activity against the mutated viruses (H5N1_V116A_ and H5N1_N295S_) was lower than that against the H5N1_wild_ virus. The activity of oseltamivir with concentration 5 µM against H5N1_wild_, H5N1_V116A_, and H5N1_N295S_ was about 90, 40, and 8.5% viral inhibition in the count of plaques, respectively. Activity of zanamivir at concentration 5 µM against the H5N1_V116A_ and H5N1_N295S_ viral inhibition in plaque count was 60 and 31%, respectively, but activity against the H5N1_wild_ and H1N1 viruses was 69.2 and 96.5% viral inhibition, respectively. The antiviral activity of the four safe concentrations of the synthetic compounds was determined by performing plaque reduction assays against the four tested viruses (H5N1_wild_, H5N1_V116A_, H5N1_N295S_, and H1N1) (Figure 6).

The results showed that the superior newly synthesized compound was **9c**, which inhibited growth of all four viruses by 52% to 97.7% at a concentration of 8.3 µM (Figure 6). The viral inhibition activity of the promising compound **9c** dramatically decreased in a dose-dependent fashion against the four tested compounds, and 6.8 µM of compound **9a** had 76% to 94% antiviral activity against the viruses (Figure 6).

#### 2.2.4. Colorimetric NA Inhibition Assay

**Figure 7:**
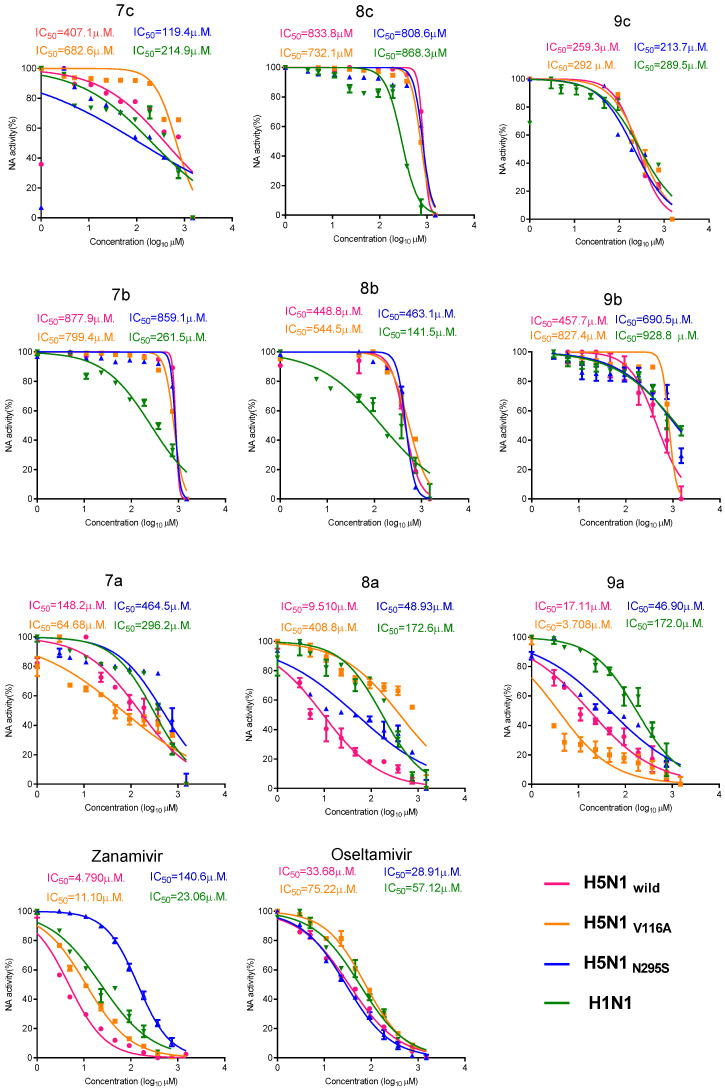
NA inhibition assay of synthetic compounds versus that of the control oseltamivir and zanamivir against the H5N1_wild_, H5N1_V116A_, H5N1_N295S_, and H1N1 viruses.

To determine the 50% neuraminidase inhibition concentration (NA IC_50_), an enzyme-linked lectin assay (ELLA) was used to determine the IC_50_ of the newly synthetic compounds through the inhibition of NA activity compared with the standard NAIs. First, we colorimetrically measured the NA activity of four tested viruses to determine the highest dilution of the virus that resulted in a maximum signal, which was then used in neuraminidase inhibition assays (Figure S1). As seen in Figure 7, the most promising compound in the benzimidazole (N) group was **9a**, followed by **8a**, which was less effective against H5N1_V116A_. The least effective compound was **7a**. The most active compound in the benzoxazole (O) group was **8b**, which was most effective against H1N1. The most active synthetic compound in the benzothiazole (S) group was **9c**, which had high anti-neuraminidase activity against the four viruses. Activity of **8c** against neuraminidase was low, and **7c** had more activity against H5N1_N295S_ and H1N1 than against H5N1_wild_ and H5N1_V116A_ (Figure 7).

Collectively, compounds with codes **9a**, **9c,** and **8b** had superior antiviral activities against the H5N1_wild_, H5N1_V116A_, H5N1_N295S_, and H1N1 viruses and were selected for further study.

#### 2.2.5. Biological Susceptibility Testing of the Developed Compounds on the Propagation of Viruses with MOI 0.005 by HA Titration

**Figure 8:**
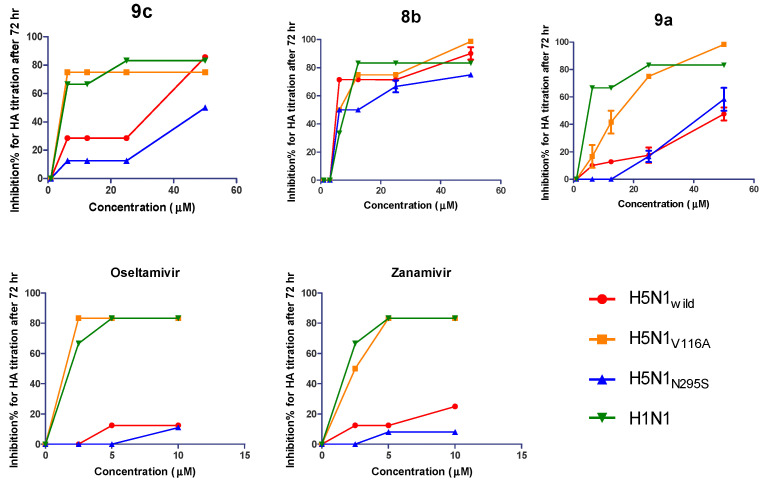
Inhibition effect of three promising synthetic compounds (**9c**, **8b**, and **9a**) on the propagation of the H5N1_wild_, H5N1_V116A_, H5N1_N295S_, and H1N1 viruses by HA titration with MOI 0.005 after 72 h.

The biological susceptibility of the superior developed compounds (**9a**, **9c**, and **8b**) on the propagation of four tested influenza viruses at a MOI of 0.005 was determined by a calculation of the reduction in the hemagglutination inhibition titer comparing four different safe concentrations (6.25, 12.5, 25, and 50 µM) with untreated and infected control cells. NAIs were included in this experiment and were tested against four viruses at different safe concentrations (2.5, 5 and 10 µM). The MDCK cells infected with the H5N1_wild_, H5N1_V116A_, H5N1_N295S_, and H1N1 viruses in the presence of the synthetic compounds showed promising inhibition in viral titers compared with the standard antiviral NAIs. Compound **9c** showed inhibition in the viral HA titer of more than 80%, with all viruses except H5N1_N295S_ showing inhibition in the HA titration of not more than 50%. Additionally, compound **8b** showed inhibition of more than 80% in the HA titration for all viruses except H5N1_N295S_ inhibition with not more than 60%; compound **9a** showed more than 80% inhibition in the HA titration for two viruses, H5N1_V116A_ and H1N1, and caused an inhibition HA titration of not more than 50% with H5N1_wild_ and H5N1_N295S_. However, the effects of zanamivir and oseltamivir showed an inhibition in the HA titration of not more than 80% against H1N1 and H5N1_V116A_ but the activity against H5N1_wild_ and H5N1_N295S_ did not exceed 20% inhibition with oseltamivir and not more than 25% with zanamivir (Figure 8).

#### 2.2.6. In Vivo Study Safety of the Synthetic Compounds

The three promising synthetic compounds (**9c**, **8b**, and **9a**) were tested for their safety in mice using three different doses (first dose, 20 mg/kg; second dose, 40 mg/kg; thirds dose, 80 mg/kg), or 1X phosphate-buffered saline (PBS) as a control, and observations were recorded daily. The mice were normal in external observations. After three days of compound or PBS administration, sera were collected to test liver and kidney functions (Table 2). The results showed that no significant differences (*p* > 0.05) were observed among the groups of mice that received the synthetic compounds and the control group except with the second dose of **9a** that affected alanine aminotransferase (ALT) and the second dose of **9c** that affected aspartate aminotransferase (AST). Hence, a 20 mg/kg dose was used to determine the antiviral efficacy of the compounds.

#### 2.2.7. Antiviral Efficacy of the New Synthetic Compounds in Mice

##### Mortality and Body Weight Loss of the Infected Mice

**Figure 9:**
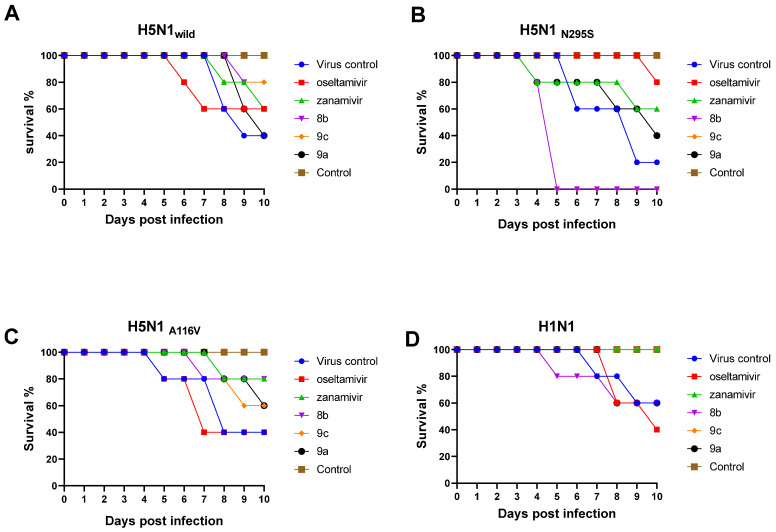
Mortality rate and antiviral efficacy of the new synthetic compounds and NAIs in infected mice. The groups were infected with different viruses and were administered synthetic compounds **8b**, **9c**, and **9a** 4 h post infection and compared with groups that were administered oseltamivir and zanamivir as drug control and compared with a viral control (VC) group, which was infected with a virus but not treated. (**A**) Study of the antiviral activity after infection with H5N1_wild_ (**B**) Study of the antiviral activity after infection with H5N1_N295S_ (**C**) Study of the antiviral activity after infection with H5N1_V116A_ (**D**) Study efficacy and antiviral activity after infection with H1N1 virus.

To examine the effect of the three superior compounds (**9a**, **9c,** and **8b**) against influenza viruses in mice, we intranasally infected 6- to 8-week-old healthy female mice with the H5N1_wild_, H5N1_V116A_, H5N1_N295S_, and H1N1 viruses. After infection, the mice were administered safe doses of the synthetic compounds (20 mg/kg) and NAIs (50 mg/kg for oseltamivir and 20 mg/kg for zanamivir). A group of untreated infected mice (virus control) as well as untreated uninfected mice (control) were included in the experiment. Mice were monitored for 10 days for weight loss (Figure S2) and mortality (Figure 9). The results indicated that the most promising compound against the H5N1_wild_ and H1N1 viruses was **9c** (Figure 9A,D). This compound achieved full protection from mortality and reduced the loss of body weight as well as zanamivir did, yielding only a 40% mortality after infection with human virus H1N1 (Figure 9D). However, the lowest antiviral activity against the H1N1 virus was with oseltamivir, which showed 60% mortality (Figure 9D); compounds **8b** and **9a** achieved 60% survival only. After infection with H5N1_wild,_ the most promising compound that could protect from mortality was **9c,** which achieved 80% survival (Figure 9A) and decreased the effects of the virus on the loss of body weight as much as zanamivir (Figure S3). Oseltamivir and **8b** achieved 60% survival only. After infection with the H5N1_V116A_, the highest antiviral efficacy was achieved by **8b**, which protected 80% of mice after infection (Figure 9); **9b** and **9c** achieved 60% survival, and oseltamivir provided only 40% survival. After infection with H5N1_N295S,_ which is more pathogenic than other viruses, oseltamivir achieved 80% survival (Figure 9B); zanamivir yielded 60% survival, compound **9c** could not protect mice from mortality, and **9a** achieved only 40% survival (Figure 9B).

##### Effect of Synthetic Compounds on a Viral Titer in a Lung and Nasal Wash

**Figure 10:**
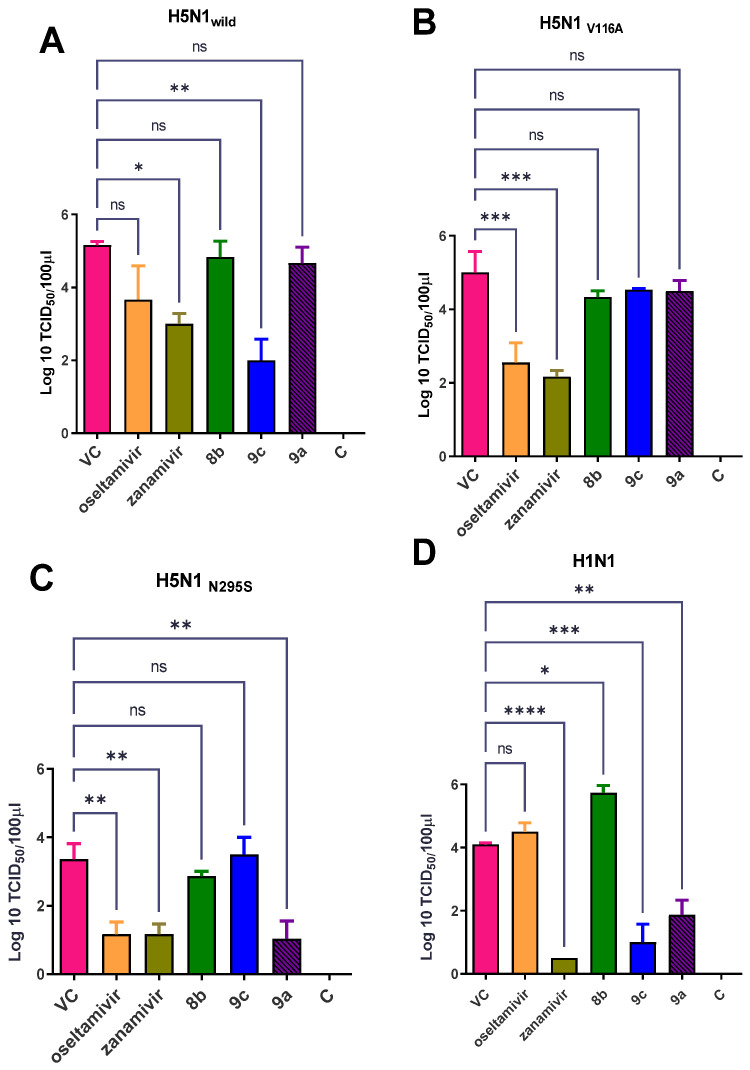
TCID_50_ titration of lung tissue after 3 DPI. (**A**) **9c** was the most promising synthetic compound after infection with H5N1_wild_ and reduced the propagation of the virus more than oseltamivir and zanamivir. (**B**) Oseltamivir and zanamivir reduced the propagation of the virus more than synthetic compounds after infection with H5N1V116A. (**C**) **9a,** oseltamivir, and zanamivir similarly reduced the propagation of the virus after infection with H5N1N295S. (**D**) **9c** and **9a** reduced the propagation of the virus after infection with H1N1 as much as zanamivir, but oseltamivir did not affect the propagation of the virus in the lung. The significant differences are indicated as follows: * *p* < 0.05, ** *p* < 0.01, *** *p* < 0.001, **** *p* < 0.0001, and ns = non-significant.

**Figure 11:**
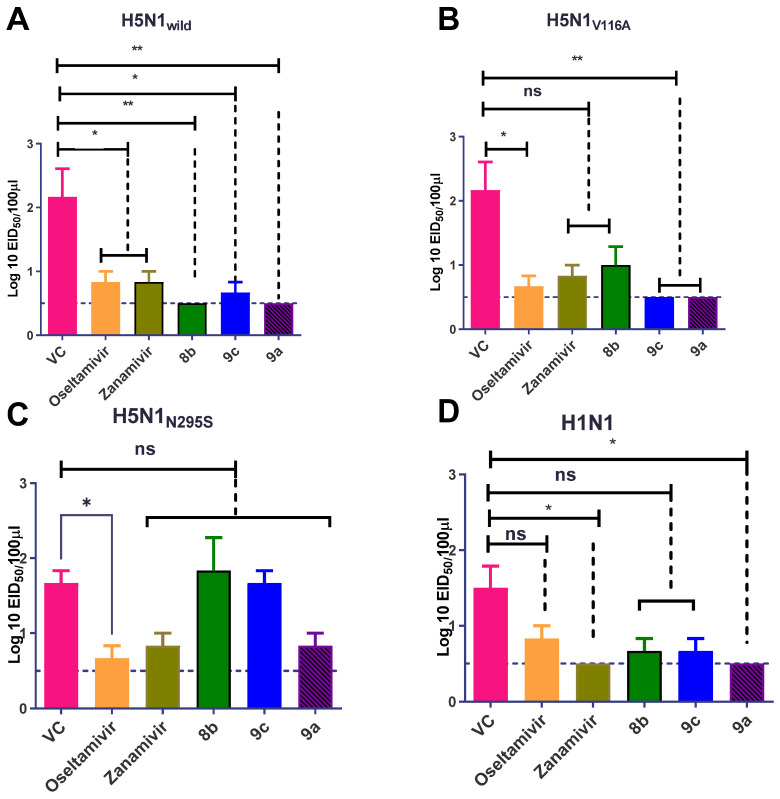
EID_50_ titration of nasal washes to detect viral shedding after 5 dpi. (**A**) Synthetic compounds reduced the viral shedding more than oseltamivir and zanamivir after infection with H5N1_wild_. (**B**) Synthetic compounds **9c** and **9a** reduced viral shedding from H5N1_V116A_-infected cells more than oseltamivir and zanamivir. (**C**) **9a** was the only synthetic compound that reduced the shedding of virus after infection with H5N1_N295S_ as well as zanamivir and oseltamivir. (**D**) Synthetic compounds reduced the viral shedding as well as zanamivir after infection with H1N1. The significant differences are indicated as follows: * *p* < 0.05, ** *p* < 0.01, and ns = non-significant.

TCID_50_ measurements after 3 DPI showed that compound **9c** reduced the propagation of the H5N1_wild_ and H1N1 viruses in lung tissue (Figure 10A,D). Zanamivir reduced the titration of four viruses with different ratios (Figure 10A–D). Oseltamivir reduced the propagation of H5N1_wild_, H5N1_V116A_, and H5N1_N295S_ but did not affect H1N1 (Figure 10A–D). Compound **9a** significantly reduced the propagation of H5N1_N295S_ and H1N1 viruses (*p* < 0.01) (Figure 10C,D). The results of the virus shedding in the nasal wash indicated that the superior compounds were **9c** and **9a**, which reduced the viral shedding of H5N1 and H5N1_V116A_ more than NAIs when the virus was detected in the nasal wash after 5 dpi (*p* < 0.01) ((Figure 11A,B). Similar to zanamivir, compound **9a** significantly reduced the viral titer of mice infected with the H1N1 virus below that of untreated infected mice after 5 dpi (*p* < 0.05) (Figure 11D). However, oseltamivir and zanamivir were more effective against the propagation of the H5N1_N295S_ virus than the synthetic compounds (Figure 11C).

### 2.3. Chemoinformatic Analysis

#### 2.3.1. Docking with Neuraminidase PDB ID: 7E6Q

**Figure 12:**
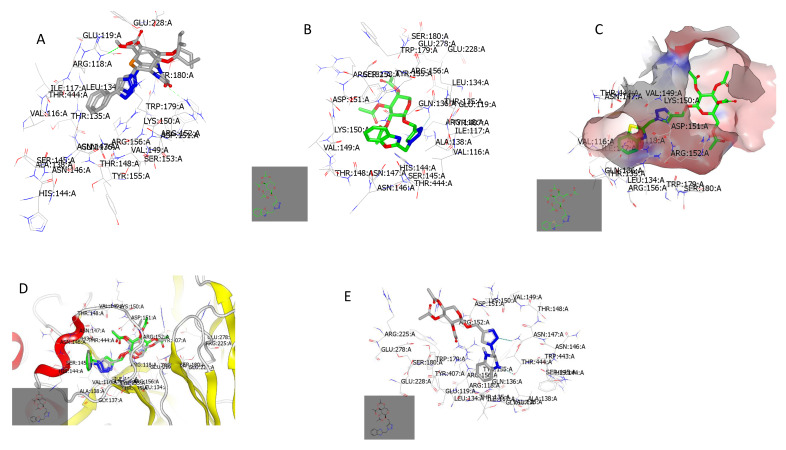
Visual representation of the docked compounds with neuraminidase (PDB ID: 7e6q). (**A**) The docked ligand (grey) overlay its co-crystalized complex (green); (**B**) Compound **9b** forms two HBs through the nitrogen atoms of its triazole moiety; (**C**) Compound **8c** displays a hydrophobic–hydrophobic interaction; (**D**) Compound **9c** (grey) completely overlays its structural isomer (green); (**E**) Compound **9a** shows a strong HB with Asn:147A and Thr:444A.

To validate the docking protocol, the co-crystalized ligand was redocked using the known experiment commands. In Figure 12A, the docked ligand (grey) showed a complete overlay with its co-crystalized complex (green). The structure formed HB with Arg: 118A and Arg: 152A. Compound **9b** formed two HBs (dotted dark blue) with Gln: 136A through the two Sp2 nitrogen molecules of the triazole moiety (Figure 12B). Compound **8c** adopted a different binding mode and pose from compound **9b** (Figure 12C). It interacted with the key amino acids without the detection of HB. The acetylated sugar was oriented peripherally from the core of the receptor but remained inside the receptor domain. Compound **9c** (grey) with an IC_50_ of 2.280 µM displayed a complete overlay with its structural isomer and there was no formation of the two HBs with Thr:135A in compound **9b** (green) with an IC_50_ of 1.679 µM, (Figure 12D). Compound **9a** displayed a unique interaction, forming a strong HB with Asn: 147A through the N2 of triazole ring and another strong HB with Thr: 444A, see Figure 12E. Among the synthesized compounds, it had a high similarity to the co-crystalized ligand.

#### 2.3.2. Ligand Efficiency and Lipophilic Efficiency Profile

Assessing the lipophilicity profile of a ligand–target interaction is a significant aspect in the drug ability of a new bioactive candidate. Currently, the justification of both molecular size and the lipophilicity (cLogP) with the drug activities (PIC_50_) designated is required. The ligand efficiency (LE) of bioactive compounds is estimated based on pIC_50_ in relation to the number of non-hydrogen atoms (NHAs) or based on the number of heavy atoms in a molecule. LE estimates the affinity of drugs based on their size instead of considering the effectiveness or binding affinity of the whole structure. LE = ΔG ÷ NHA or LE = (pIC_50_ × 1.37) ÷ NHA; where ΔG = Gibb’s free energy, IC_50_ = half maximal inhibitory concentration (in terms of molar concentration), and NHA = non-hydrogen atom. The challenge in the drug ability of new drug candidates is in increasing the activity while keeping the lipophilicity constant to avoid any “molecular obesity” during the drug optimization process. Compounds **9a**–**c** had LE values that were better than compounds **8b** and **8c** (Table 3).

The other parameter of lipophilicity is the ligand lipophilic efficiency (LLE). LLE is a way of calculating the affinity of a bioactive compound with respect to its lipophilicity. LLE is the difference between the potency and Clog P based on the following equation:

LLE = pIC50 − cLogP

The compounds in Table 3 displayed LLE values of 6.43–7.32. An LLE value ≥ 5 is recommended for drug candidates.

## 3. Discussion

Although neuraminidase drug resistance among strains—especially N1, which has several mutations—of circulating influenza A viruses is increasing and the emergence of NAI resistance has become common, targeting viral NA is ideal because it is a surface glycoprotein, making it more accessible to drugs. In order to develop more potent NA inhibitors, the use of certain structural features of available drugs could be a rapid approach towards overcoming viral resistance. A click chemistry approach was used to synthesize targeted glycosyl-1,2,3-triazoles incorporating different acetylated cyclic sugar moieties. The required azide derivatives were synthesized according to previously reported methods [34,35,36,37,38]. The acetylated terminal acetylenic glycoside derivatives **4**–**6** were reacted via applying click conditions with the azides **1**–**3,** resulting in the formation of the disubstituted 1,2,3-triazole glycosides **7**–**9** in good yields. The synthetic strategy was based on the application of an organic solvent soluble halogenated Cu (I) complex incorporating a triphenyl phosphine ligand synthesized via a modified procedure, which resulted in a higher catalyst yield in a shorter reaction time. *Iso*-Butanol has been shown to be the more efficient solvent in these click reactions, under the effect of the previous catalytic system, in the study of different solvent systems, either single or mixed, such as an isopropanol and n-butanol-water mixture. An advantage of the applied procedure is that the in situ reduction of Cu(I) salts by a reducing material such as ascorbate is not needed because such catalysts incorporate the required Cu(I) species. Although the reaction was complete and resulted in the formation of single products, purification using SiO_2_ column chromatography with ethyl acetate/methanol was carried out to obtain the targeted pure triazolyl glycosides.

The IR spectra of the produced hetero-aryl hybrid glycosides **7**–**9** showed the presence of the absorption bands of the acetyl carbonyl groups at their characteristic regions. Furthermore, the IR spectra revealed the disappearance of the characteristic absorption band corresponding to the azide group in the reacting hetero-aryl azide compounds. The ^1^H NMR spectra of the acetylated 1,2,3-triazoly-*N*-glycosides **7**–**9** displayed the signals assigned to glycosyl part protons, including those of the acetyl methyl groups, in addition to the signals of remaining protons of the sugar moiety. The observed coupling value (*J*-constant) of the anomeric proton (*H*-1 in the glycopyranosyl moiety), displayed as a doublet signal at 9.4–10.2 Hz accounts for the *β*-conformation nature of the attachment of the glycosyl part to the 1,2,3-triazole ring. Their ^13^C NMR spectra also confirmed the assigned structures, and the signals of the carbons in the assigned structures appeared at their expected characteristic regions. The newly synthesized compounds showed antiviral activity against the H1N1, H5N1_wild_, H5N1_V116A_, and H5N1_N295S_ viruses in the inhibition neuraminidase enzyme assays where the activity was compared to the activity of neuraminidase inhibitors as drug controls in the same condition of assays to improve the efficacy of drugs so they can be active against different strains of influenza A, including sensitive and resistant strains, according to the type of mutation present in the virus, and yield products that are more stable in the body with different routes of administrations. The current study showed numerous novel outcomes to be used as alternatives to anti-influenza drugs. Both **9c** and **9a** had better antiviral activity and safety profiles in vivo than oseltamivir and had activity and safety profiles similar to those of zanamivir. These compounds are suitable for administration in other forms, unlike zanamivir, which is administered only in inhalation form, is unsuitable for children, and causes some side effects making it unsuitable for patients with underlying airways disease, bronchospasm, and allergic-like reactions. Compound **9c,** a benzothiazole compound, had a promising effect against H1N1 and H5N1 in vitro and in vivo. Benzoxazole, the aromatic organic compound used in this study in combination with carbohydrates to achieve anti-influenza activity by inhibiting neuraminidase enzyme activity, had promising effects against rg H5N1_V116A_ and a mild effect against wild-type H5N1 in vitro and in vivo.

Benzoxazole is already in some drugs on the market such as flunoxaprofen, calcimycin, benoxaprofen, chloroxazol, and boxazomycin B. In vitro studies were conducted to use Benzoxazole as an antimicrobial against Gram-positive bacteria, Gram-negative bacteria, and the fungal strains *Candida albicans* and *Aspergillus niger*, and its performance was compared to that of ofloxacin and fluconazole. The determination of in vitro anticancer activity (IC_50_ value) was completed by a sulforhodamine B assay on a human colorectal carcinoma (HCT116) cancer cell line using 5-fluorouracil as the standard drug [28]. Benzothiazole, with a fused heterocyclic moiety, is a valuable scaffold with diverse biological activities, such as anticancer, anti-inflammatory, antimicrobial, antiviral, antimalarial, and anticonvulsant effects [27]. Compound **9a**, a derivative of Benzimidazole, is a heterocyclic aromatic organic compound that exhibits a similarly wide range of diverse and pharmaceutically useful activities [29]. In this study, benzimidazole (**9a**) was combined with three different forms of carbohydrates to form a compound targeting the neuraminidase enzyme in the influenza A virus; **9a** had antiviral activity against H1N1, rg H5N1_N295S_, and wild-type H5N1 and the variation in response presented due to the variation in genetic material for each strain led to a different interaction with the compounds.

The docking analysis and lipophilicity parameter calculations demonstrated that the glycosylated compounds with a d-xylose moiety had the best activity and will allow the design and synthesis of drug candidates. Limitations of the study are that the in vitro activity of the promising compounds in human lung epithelial cells and metabolic stability studies was not analyzed, and the antiviral activity against other human influenza viruses, such as influenza A subtype H3N2 and influenza B viruses, was not examined. Finally, multidisciplinary collaboration is necessary to accelerate the development of new targeted drugs. We recommend further preclinical and clinical studies of these promising compounds to find alternative therapeutic antiviral agents targeting NAIs in the near future.

## 4. Materials and Methods

### 4.1. Chemistry

The melting points were measured by using a melting point apparatus and were not corrected. Infrared spectra (KBr for solid or neat for liquid) were measured on a Bruker-Vector 22. ^1^H NMR and ^13^C NMR spectra were obtained by using a JEOL EX-500 MHz spectrometer and (CDCl_3_) or DMSO-d_6_, with TMS as an internal standard (Figure S4). Chemical shifts are quoted in δ and were related to those of the solvents. Splitting patterns were designated as follows: s, singlet, and m, multiple. Elemental analyses were operated using a Mario Elemental apparatus. Thin-layer chromatography (TLC) was used to mentor all reactions. The benzoxazole, benzimidazole, and benzothiazole azides **3a**–**c** were prepared by the reaction of their corresponding halide compounds **2a**–**c** with sodium azide according to previously reported procedures [34,35,36,37,38].

#### 4.1.1. 2-Propynyl 2,3,4,6-Tetra-*O*-acetyl-β-d-glucopyranosides (**4**–**6**)

The terminal acetylenic sugars were prepared according to the procedure of Mereyala and Gurrala [40]. Thus, to a solution of β-d-glucopyranose pentaacetate, β-d-galactopyranose pentaacetate, or β-d-xylopyranose pentaacetate (25.6 mmol) in dichloromethane (200 mL) was added freshly distilled propargyl alcohol (1.8 mL, 30.7 mmol) and BF_3_.Et_2_O (4.8 mL, 38.4 mmol) at 0 °C, and the reaction mixture was stirred at room temperature for 2 h. After completion of the reaction, anhydrous K_2_CO_3_ (4.8 g) was added, and stirring was continued for a further 30 min. The reaction mixture was filtered and washed with dichloromethane. The filtrate was washed with water (2 × 150 mL), the aqueous phase was separated and extracted with dichloromethane (2 × 50 mL), and the combined organic phases were dried (Na_2_SO_4_) and concentrated to yield a solid that was crystallized (dichloromethane-hexane) to obtain the title compound **1c** (9.10 g, 92%) as a crystalline solid.

#### 4.1.2. Bromo-tris-(triphenylphosphine) Copper (I) (CuL3Br)

This Cu(I) complex was synthesized via a slight modification of a published procedure [26]. Triphenylphosphine (4.89 g, 20 mmol) was dissolved in hot methanol (140 mL) in a 250 mL round-bottom flask equipped with a Teflon stir bar. Cu (II) bromide (1.12 g, 5 mmol) was added in small portions to the previous solution. The resulting mixture was refluxed with stirring for 1 h, and the flask was allowed to cool to an ambient temperature. The white precipitate was then filtered via a Buchner funnel to isolate the desired compound as a white solid. After washing repeatedly with methanol and then diethyl ether, the resultant white solids were dried under reduced pressure to give 4 g (88%, based on CuBr_2_) of product with a melting point (m.p.) of 166–168 °C (lit. 167 °C) [6].

#### 4.1.3. General Procedure for the Cu(I)-Catalyzed Click Dipolar Coupling of Benzohetero-Azides and Terminal Acetylenic Pyranosyl Sugars

The azide derivatives **3a**–**c** (1.2 mmol), the corresponding acetylenic per-*O*-acetylated sugar (1 mmol), and CuL_3_Br (10 mol%) were mixed successively in 2-butanol (25 mL), and the reaction mixture was stirred at room temperature for 48 h. After completion of the reaction as indicated by TLC, the solvent was removed under reduced pressure, and the remaining residue was purified via SiO_2_ column chromatography using ethyl acetate/methanol (9.5:0.5) to prepare the 1,2,3-triazole glycoside derivatives **7**–**9**.

#### 4.1.4. (2*R*,3*R*,4*S*,5*R*,6*R*)-2-((1-((1*H*-Benzo[d]imidazol-2-yl)methyl)-1*H*-1,2,3-triazol-4-yl)methoxy)-6-(acetoxymethyl)tetrahydro-2*H*-pyran-3,4,5-triyl Triacetate (**7a**)

Compound **7a** was synthesized from compounds **3a** and **4** following the general procedure. Pale yellow foam, Yield 84%. IR (KBr) υ (cm^−1^): 3442 (NH), 3145 (CH-arom.), 2961 (CH-aliph.), 1751 (C = O), 1605 (C = C). ^1^H NMR (CDCl_3_) δ/ppm: 1.89, 1.95, 1.99, 2.03 (4s, 12H, 4CH_3_), 3.63–3.67 (m, 1H, H-5′), 4.12–1.14 (dd, 1H, *J* = 10.8, 3.2 Hz, H-6′), 4.18–4.20 (dd, 1H, *J* = 8.6, 3.2 Hz, H-6″), 4.63–4.66 (dd, 1H, *J* = 8.8, *J* = 8.2 Hz, H-4′), 4.88–5.07 (m, 3H, CH_2_, H-2′), 5.17–5.20 (t, 1H, *J* = 8.2 Hz, H-3′), 5.79 (d, 1H, *J* = 9.8 Hz, H-1′), 5.82 (s, 2H, CH_2_), 7.32–7.34 (m, 1H, Ar-H), 7.41–7.50 (m, 2H, Ar-H), 7.59–7.67 (m, 1H, Ar-H), 7.81 (s, 1H, triazole-H), 9.98 (br, 1H NH). Analysis Calcd. for (C_25_H_29_N_5_O_10_): C, 53.67; H, 5.22; N, 12.52. Found: C, 53.41; H, 5.17; N, 12.69.

#### 4.1.5. (2*R*,3*R*,4*S*,5*R*,6*R*)-2-(Acetoxymethyl)-6-((1-(benzo[d]oxazol-2-ylmethyl)-1*H*-1,2,3-triazol-4-yl)methoxy)tetrahydro-2*H*-pyran-3,4,5-triyl Triacetate (**7b**)

Compound **7b** was synthesized from compounds **3b** and **4** following the general procedure. Pale brown thick oil, Yield 75%. IR (KBr) υ (cm^−1^): 2923 (CH-aliph.), 3095 (CH-arom.), 1752 (C = O), 1605 (C = C). ^1^H NMR (CDCl_3_) δ/ppm: 1.85, 1.92, 1.94, 2.07 (4s, 12H, 4CH_3_), 3.65 (m, 1H, H-5′), 4.09–4.12 (dd, 1H, *J* = 3.8, *J* = 11.2 Hz, H-6′), 4.15–4.17 (m, 1H, H-6″),4.62 (dd, 1H, *J* = 8.1, *J* = 7.8 Hz, H-4′), 4.80–4.91 (m, 3H, CH_2_, H-2′), 5.09–5.13 (t, 1H, *J* = 7.8 Hz, H-3′), 5.79 (d, 1H, *J* = 9.6 Hz, H-1′), 5.81 (s, 2H, CH_2_), 7.28–7.29 (m, 1H, Ar-H), 7.30–7.46 (m, 2H, Ar-H), 7.59–7.62 (m, 1H, Ar-H), 7.65 (s, 1H, triazole-H). Analysis Calcd. for (C_25_H_28_N_4_O_11_): C, 53.57; H, 5.04; N, 10.00. Found: C, 53.38; H, 5.09; N, 9.88.

#### 4.1.6. (2*R*,3*R*,4*S*,5*R*,6*R*)-2-(Acetoxymethyl)-6-((1-(benzo[d]thiazol-2-ylmethyl)-1*H*-1,2,3-triazol-4-yl)methoxy)tetrahydro-2*H*-pyran-3,4,5-triyl Triacetate (**7c**)

Compound **7c** was synthesized from compounds **3c** and **4** following the general procedure. Pale brown thick oil, Yield 73%. IR (KBr) υ (cm^−1^): 3060 (CH-arom.), 2957 (CH-aliph.), 1755 (C = O), 1608 (C = C). ^1^H NMR (CDCl_3_) δ/ppm: 1.79, 1.88, 1.92, 1.96 (4s, 12H, 4CH_3_), 3.61-3.70 (m, 1H, H-5′), 4.02–4.06 (dd, 1H, *J* = 10.8, 3.2 Hz, H-6′), 4.13–4.14 (dd, 1H, *J* = 3.2, 8.4 Hz, H-6″), 4.60 (d, 1H, *J* = 7.8 Hz, H-4′), 4.77–4.93 (m, 3H, CH_2_, H-2′), 5.10–5.12 (t, 1H, *J* = 8.4 Hz, H-3′), 5.85 (d, 1H, *J* = 10.2 Hz, H-1′), 5.88 (s, 2H, CH_2_), 7.32-7.41 (m, 2H, Ar-H), 7.74 (s, 1H, triazole-H), 7.76–7.79 (m, 1H, Ar-H), 7.93 (d, 1H, *J* = 9 Hz, Ar-H). Analysis Calcd. for (C_25_H_28_N_4_O_10_S): C, 52.08; H, 4.90; N, 9.72. Found: C, 51.93; H, 4.79; N, 9.83.

#### 4.1.7. (2*R*,3*R*,4*S*,5*S*,6*R*)-2-((1-((1*H*-Benzo[d]imidazol-2-yl)methyl)-1*H*-1,2,3-triazol-4-yl)methoxy)-6-(acetoxymethyl)tetrahydro-2*H*-pyran-3,4,5-triyl Triacetate (**8a**)

Compound **8a** was synthesized from compounds **3a** and **5** following the general procedure. Pale brown thick oil, Yield 82%. IR (KBr) υ (cm^−1^): 3290 (NH), 3095 (CH-arom.), 2960 (CH-aliph.), 1750 (C = O), 1602 (C = C). ^1^H NMR (CDCl_3_) δ/ppm: 1.82, 1.89, 1.95, 1.98 (4s, 12H, 4CH_3_), 3.51–3.54 (m, 1H, H-5′), 3.97–4.00 (dd, 1H, *J* = 10.8, 3.2 Hz, H-6′), 4.11–4.14 (dd, 1H, *J* = 8.6, 3.2 Hz, H-6″), 4.64–4.66 (dd, 1H, *J* = 8.8, *J* = 8.2 Hz, H-4′), 4.72–4.98 (m, 4H, 2CH_2_), 5.03-5.06 (m, 1H, H-2′), 5.22 (t, 1H, *J* = 9.6 Hz, H-3′), 5.80 (d, 1H, *J* = 9.6 Hz, H-1′), 7.17–7.21 (m, 1H, Ar-H), 7.55–7.58 (m, 2H, Ar-H), 7.59 (m, 1H, Ar-H), 8.01 (s, 1H, triazole-H), 8.23 (br, 1H, NH). Analysis Calcd. for (C_25_H_29_N_5_O_10_): C, 53.67; H, 5.22; N, 12.52. Found: C, 53.79; H, 5.29; N, 12.59.

#### 4.1.8. (2*R*,3*S*,4*S*,5*R*,6*R*)-2-(Acetoxymethyl)-6-((1-(benzo[d]oxazol-2-ylmethyl)-1*H*-1,2,3-triazol-4-yl)methoxy)tetrahydro-2*H*-pyran-3,4,5-triyl Triacetate (**8b**)

Compound **8b** was synthesized from compounds **3b** and **5** following the general procedure. Pale brown thick oil, Yield 69%. IR (KBr) υ (cm^−1^): (CH-aliph.), 3105 (CH-arom.), 1752 (C = O), 1605 (C = C). ^1^H NMR (CDCl_3_) δ/ppm: 1.88, 1.94, 1.99, 2.02 (4s, 12H, 4CH_3_), 3.65-3.68 (m, 1H, H-5′), 4.12–1.14 (dd, 1H, *J* = 10.8, 3.2 Hz, H-6′), 4.18-4.19 (dd, 1H, *J* = 8.6, 3.2 Hz, H-6″), 4.64–4.66 (dd, 1H, *J* = 8.8, *J* = 8.2 Hz, H-4′), 4.83–4.96 (m, 3H, CH_2_, H-2′), 5.25 (t, 1H, *J* = 8.2 Hz, H-3′), 5.80 (d, 1H, *J* = 9.8 Hz, H-1′), 5.84 (s, 2H, CH_2_), 7.32–7.34 (m, 1H, Ar-H), 7.41–7.50 (m, 2H, Ar-H), 7.59–7.67 (m, 1H, Ar-H), 7.81 (s, 1H, triazole-H); ^13^C NMR (CDCl_3_) δ/ppm: 20.42, 20.57, 47.04, 61.67, 62.72, 68.23, 71.08, 71.79, 72.65, 99.78, 110.88, 120.42, 123.66, 124.93, 126.03, 128.35, 128.51, 131.90, 132.01, 169.30, 170.03, 170.51. Analysis Calcd. for (C_25_H_28_N_4_O_11_): C, 53.57; H, 5.04; N, 10.00. Found: C, 53.32; H, 5.01; N, 10.09.

#### 4.1.9. (2*R*,3*S*,4*S*,5*R*,6*R*)-2-(Acetoxymethyl)-6-((1-(benzo[d]thiazol-2-ylmethyl)-1*H*-1,2,3-triazol-4-yl)methoxy)tetrahydro-2*H*-pyran-3,4,5-triyl Triacetate (**8c**)

Compound **8c** was synthesized from compounds **3c** and **5** following the general procedure. Pale brown thick oil, Yield 78%. IR (KBr) υ (cm^−1^): 3064 (CH-arom.), 2938 (CH-aliph.), 1755 (C = O), 1604 (C = C). ^1^H NMR (CDCl_3_) δ/ppm: 1.84, 1.93, 1.96, 1.99 (4s, 12H, 4CH_3_), 3.68–3.71 (m, 1H, H-5′), 4.10-4.12 (dd, 1H, *J* = 10.8, *J* = 2.8 Hz, H-6′), 4.15–4.17 (dd, 1H, *J* = 10.8, *J* = 3.1 Hz, H-6″), 4.63 (dd, 1H, *J* = 8.1, *J* = 6.8 Hz, H-4′), 4.81–4.99 (m, 5H, 2CH_2_, H-2′), 5.10 (t, 1H, *J* = 8.2 Hz, H-3′), 5.24 (d, 1H, *J* = 9.6 Hz, H-1′), 7.37–7.49 (m, 1H, Ar-H), 7.56–2.65 (m, 1H, Ar-H), 7.76 (s, 1H, triazole-H), 7.82 (d, *J* = 7.8 Hz, 1H, Ar-H), 7.98 (d, *J* = 8.1 Hz, 1H, Ar-H); ^13^C NMR (CDC_l3_) δ/ppm: 30.39, 20.43, 20.58, 22.63, 51.01, 51.43, 61.69, 62.68, 68.22, 71.07, 71.81, 72.65, 99.72, 121.78, 123.46, 125.97, 126.58, 128.31, 128.47, 131.85, 131.90, 132.03, 133.07, 135.41, 152.67, 162.94, 169.19, 169.27, 170.03, 170.48. Analysis Calcd. for (C_25_H_28_N_4_O_10_S): C, 52.08; H, 4.90; N, 9.72; Found: C, 51.90; H, 4.94; N, 9.81.

#### 4.1.10. (2*R*,3*R*,4*S*,5*R*,)-2-((1-((1*H*-Benzo[d]imidazol-2-yl)methyl)-1*H*-1,2,3-triazol-4-yl)methoxy)tetrahydro-2*H*-pyran-3,4,5-triyl Triacetate (**9a**)

Compound **9a** was synthesized from compounds **3a** and **6** following the general procedure. Pale brown thick oil, Yield 78%. IR (KBr) υ (cm^−1^): 3461 (NH), 3061 (CH-arom.), 2951 (CH-aliph.), 1755 (C = O), 1600 (C = C). ^1^H NMR (CDCl_3_) δ/ppm: 1.92, 1.95, 1.99 (3s, 9H, 3CH_3_), 4.10–4.12 (dd, 1H, *J* = 3.6, *J* = 10.8 Hz, H-5′), 4.16–4.18 (dd, 1H, *J* = 10.6, *J* = 2.8 Hz, H-5″), 4.62–4.65 (dd, 1H, *J* = 6.8, *J* = 8.8 Hz, H-4′), 4.81–4.97 (m, 3H, CH_2_, H-2′), 5.24 (t, 1H, *J* = 7.8 Hz, H-3′), 5.81 (s, 2H, CH_2_), 5.84 (d, 1H, *J* = 10.2 Hz, H-1′), 7.29–7.32 (m, 1H, Ar-H), 7.40-7.46 (m, 2H, Ar-H), 7.56–7.66 (m, 1H, Ar-H), 7.80 (s, 1H, triazole-H), 9.05 (br, 1H, NH). Analysis Calcd. for (C_22_H_25_N_5_O_8_): C, 54.21; H, 5.17; N, 14.37. Found: 54.37; H, 5.11; N, 14.52.

#### 4.1.11. (2*R*,3*R*,4*S*,5*R*)-2-((1-(Benzo[d]oxazol-2-ylmethyl)-1*H*-1,2,3-triazol-4-yl)methoxy)tetrahydro-2*H*-pyran-3,4,5-triyl Triacetate (**9b**)

Compound **9b** was synthesized from compounds **3b** and **6** following the general procedure. Pale brown thick oil, Yield 71%. IR (KBr) υ (cm^−1^): 3065 (CH-arom.), 2941 (CH-aliph.), 1755 (C = O), 1605 (C = C). ^1^H NMR (CDCl_3_) δ/ppm: 1.98, 2.02, 2.06 (3s, 9H, 3CH_3_), 3.75–3.81 (dd, 1H, *J* = 11.2, *J* = 3.4 Hz, H-5′), 4.05–4.08 (m, 1H, H-5″), 4.62–4.66 (m, 1H, H-4′), 4.90 (s, 2H, CH_2_), 4.95–4.99 (dd, 1H, *J* = 7.8 Hz, *J* = 9.6 H-2′), 5.23 (t, 1H, *J* = 7.8 Hz, H-3′), 5.80 (s, 2H, CH_2_), 5.84 (d, 1H, *J* = 9.6 Hz, H-1′), 7.34–7.37 (m, 1H, Ar-H), 7.44–7.55 (m, 2H, Ar-H), 7.61–7.68 (m, 2H, Ar-H). Analysis Calcd. for (C_22_H_24_N_4_O_9_): C, 54.10; H, 4.95; N, 11.47. Found: C, 54.24; H, 4.91; N, 11.37.

#### 4.1.12. (2*R*,3*R*,4*S*,5*R*)-2-((1-(Benzo[d]thiazol-2-ylmethyl)-1*H*-1,2,3-triazol-4-yl)methoxy)tetrahydro-2*H*-pyran-3,4,5-triyl Triacetate (**9c**)

Compound **9c** was synthesized from compounds **3c** and **6** following the general procedure. Pale brown thick oil, Yield 75%. IR (KBr) υ (cm^−1^): 3064 (CH-arom.), 2932 (CH-aliph.), 1752 (C = O), 1603 (C = C). ^1^H NMR (CDCl_3_) δ/ppm: 1.90, 1.98, 2.01 (3s, 9H, 3CH_3_), 4.05–4.08 (dd, 1H, *J* = 3.4, *J* = 10.6 Hz, H-5′), 4.11–4.13 (dd, 1H, *J* = 10.6, *J* = 2.8 Hz, H-5″), 4.61 (dd, 1H, *J* = 6.6 Hz, H-4′), 4.78–4.91 (m, 3H, CH_2_, H-2′), 5.24 (t, 1H, *J* = 8.2 Hz, H-3′), 5.90 (s, 2H, CH_2_), 5.94 (d, 1H, *J* = 9.6 Hz, H-1′), 7.41–7.52 (m, 1H, Ar-H), 7.61–7.65 (m, 1H, Ar-H), 7.75 (s, 1H, triazole-H), 7.85 (d, 1H, *J* = 7.8 Hz, Ar-H), 8.02 (d, 1H, *J* = 8.1 Hz, Ar-H). Analysis Calcd. for (C_22_H_24_N_4_O_8_S): C, 52.38; H, 4.80; N, 11.11. Found: C, 52.19; H, 4.85; N, 11.27.

### 4.2. Cells, Wild-Type Viruses, and Antiviral Compounds

Madin–Darby canine kidney (MDCK) cells (ATCC) were maintained in Dulbecco’s Modified Eagle’s Medium (DMEM) (Lonza, Basel, Germany) supplemented with 10% of inactivated fetal bovine serum (FBS) (Gibco, Waltham, MA, USA) and 1% of a mixture of antibiotic–antimycotic (Gibco) and grown at 37 °C in 5% CO_2_.

The highly pathogenic A/chicken/Egypt/B13825A/2017 (H5N1_wild_, clade 2.2.1.2) and influenza A virus A/California/04/2009 (H1N1, H1N1pdm09) were used in this study. Both viruses were propagated in 11-day-old specific pathogen-free embryonated chicken eggs (SPF-ECEs) for 48 h. The propagated viruses were titrated by hemagglutination (HA) assay, 50% tissue culture infectious dose assay (TCID_50_/mL), and 50% egg infectious dose assay (EID_50_/mL).

Oseltamivir carboxylate (Roche Diagnostics GmbH, Mannheim, Germany)) and zanamivir (St. Louis, MO, USA) were used as control NAIs.

### 4.3. Generation of NAI-Resistant H5N1 Viruses

Mutations conferring reduced neuraminidase inhibitor activity (V116A and N295S) were individually introduced into the NA segment of the A/chicken/Egypt/ B13825A/2017(H5N1) virus by using specific mutagenesis primers and a QuikChange site-directed mutagenesis kit (Agilent, Santa Clara, CA, USA) according to manufacturer instructions. Sanger sequencing was performed to confirm the introduction of these mutations. Reverse genetics was used to generate the mutated H5N1_V116A_ and H5N1_N295S_ viruses using 6 internal segments (PB2, PB1, PA, NP, M, NS) of the A/Puerto Rico/8/34 (H1N1) virus and HA and NA from the A/chicken/Egypt/B13825A/2017(H5N1) virus. The rescued viruses (H5N1_V116A_ and H5N1_N295S_), H5N1_wild_, and H1N1 were propagated in allantoic cavities of SPF-ECEs for 48 h followed by titration by plaque titration assay, EID_50_, and TCID_50_.

### 4.4. MTT Assay

To determine the half-maximal cytotoxic concentration (CC_50_) of each tested compound in the MDCK cells, the 3-(4,5-Dimethylthiazol-2-yl)-2,5diphenyltetrazolium bromide (MTT) assay was conducted as previously described [41], with minor modifications. Briefly, a broad range of serial concentrations of the tested compounds were prepared in DMEM from 15 Mm to 3 µM. MDCK cells were treated with various concentrations of the tested compounds in triplicates. After incubation at 37 °C for 72 h, the cells were washed with 1× PBS-balanced salt solution, and 20 μL of MTT solution (5 mg/mL) was added to each well. Then the cells were incubated at 37 °C for three hours to allow the violet formazan crystals to form. The formed crystals were dissolved in dimethyl sulfoxide (Sigma), and the absorbance of the formed color was measured at λmax 540 nm, with 620 nm as a reference wavelength. The CC_50_ values for each tested compound were calculated via nonlinear regression analysis in GraphPad Prism software version 5.01 (San Diego, CA, USA).

### 4.5. Crystal Violet Assay

Antiviral activity of the compounds was tested according to the procedure previously described [39]. The 50% inhibitory concentration (IC_50_) value for each tested compound was calculated via nonlinear regression analysis in GraphPad Prism software.

### 4.6. Plaque Reduction Assay

To assess the preliminary antiviral activity of the compounds, plaque reduction assays were carried out in cultured MDCK cells in six-well plates according to the procedure previously described [42].

### 4.7. Colorimetric NA Inhibition Assay

The inhibition of NA activity by the tested compounds was measured via enzyme-linked lectin assay (ELLA). To measure the NA IC_50_, serial dilutions of the tested compounds were mixed with standardized virus dilutions and incubated at 37 °C for 18 h on fetuin-coated plates. NA IC_50_ values for each tested compound were calculated by performing a nonlinear regression analysis in GraphPad Prism software (version 5.01).

### 4.8. Biological Susceptibility Testing of the Developed Compounds

The biological susceptibility of the developed compounds for the H5N1wild, H5N1_V116A_, H5N1_N295S_, and H1N1 viruses was ascertained. In brief, monolayers of MDCK cells were cultured before the day of infection. The viruses were diluted to reach 0.005 multiplicity of infection (MOI) and incubated with 6.25, 12.5, 25, and 50 µM safe doses of the tested compounds for 1 h. The medium was discarded from the MDCK cells. The cells were washed twice with 1X PBS. A mixture of each virus at MOI 0.005 and safe concentrations of the tested compounds were added to MDCK cells. The virus control (without any treatment) was added to each plate. Cells were incubated at 37 °C under 5% CO_2_ for 1 h, with hand rocking every l5 min to ensure a homogenous exposure of the cells to infection and to avoid drying. After 1 h, the infection medium was added to the cells and incubated at 37 °C under 5% CO_2_ for 72 h. Next, 300 µL of the media was collected every 24 h for viral detection and titration by HA assay. The calculation follows the below equation:

Percentage of hemagglutination titer inhibition = hemagglutination titer of virus control untreated − hemagglutination titer of treated virus/hemagglutination titer of virus control untreated × 100.

### 4.9. Animal Experiments: In Vivo Study of the Synthetic Compounds’ Safety

Superior compounds showing antiviral activity in vitro were subjected to in vivo studies. Compounds **8b**, **9c**, and **9a** were dissolved in deionized distilled water with 10% DMSO; five C57BL/6 female mice (6 to 8 weeks old) for each group were administered 3 different doses (1st dose, 20 mg/kg; 2nd dose, 40 mg/kg; 3rd dose, 80 mg/kg), and observations were recorded daily. Loss of body weight and mortality for seven days among the oseltamivir, zanamivir, and control group of mice were compared after the administration of 50 mg/kg for oseltamivir [43], 20 mg/kg for zanamivir [44], and 1× phosphate-buffered saline (PBS) for the control group. After three days of compound administration, sera were collected to test liver and kidney functions and compared with those of the control group, which was administered 1X PBS.

Based on the results of prior experiments, a volume of 25 µL (20 mg/kg) of **8b**, **9c**, and **9a** was administered once daily for 5 days by inhalation after 4 h of infection with each tested virus. We recommended this route of administration, similar to that of zanamivir, which was a co-crystallized ligand in the docking study. C57BL/6 mice were divided into 6 groups for each virus (oseltamivir, zanamivir, **8b**, **9c**, **9a**, and virus control), each group consisting of 8 female mice (6 to 8 weeks old). Viruses were H5N1_wild_, H5N1_V116A_, H5N1_N295S_, and H1N1. The mice were anesthetized via isoflurane inhalation and intranasally inoculated with influenza virus in PBS. H1N1 was inoculated according to TCID_50_ in a 50-µL volume. H5N1 inoculations were determined according to EID_50_, with the H5N1_wild_ and H5N1_V116A_ viruses given in a 20 µL volume and the H5N1_N295S_ virus given in a 30 µL volume for each mouse. An uninfected control group was anesthetized and intranasally inoculated with 20 µL of 1X PBS. Five mice per group were monitored for 10 days post infection (dpi) for body weight loss and mortality. Mortality was recorded as death or loss of 30% of body weight (the threshold at which animals were euthanized). Three mice per group were euthanized at 3 dpi. Lungs were collected. To determine viral titers, 0.1 g of each lung was homogenized in 0.9 mL 1X PBS by using a Qiagen Tissue Lyser II (Qiagen, Hilden, Germany). Organ homogenates were centrifuged at 2000 rpm for 5 min, and the virus titer in the supernatants was determined by TCID_50_/100 µL as discussed before. Nasal washes were collected at 5 dpi to study virus shedding via EID_50_ titration.

Neuraminidase inhibitors such as oseltamivir (Tamiflu; Roche Laboratories, Nutley, NJ, USA) were used as drug controls; mice were administered 30 µL (50 mg/kg) oseltamivir once daily for 6 days by oral gavage, and treatment was started 24 h prior to infection [43]. Mice were administered 25 µL (20 mg/kg) of zanamivir (Relenza; GlaxoSmithKline, Brentford, UK) once daily for 5 days by inhalation 4 h after infection [44].

### 4.10. Chemoinformatic Studies

#### 4.10.1. Molecular Docking

The X-ray crystal structure coordinates of neuraminidase (PDB ID: 7e6q) were retrieved from PDB with its co-crystallized bound ligand. The docking study was performed by using OpenEye Scientific Software version 2.2.5 (Academic license 2021, Yaseen Elshaier lab, Santa Fe, NM, USA, http://www.eyesopen.com, accessed in 1 December 2021). To validate the docking study, the co-crystal bound ligand was redocked.

#### 4.10.2. Physiochemical Parameter and Lipophilicity Calculations

Compound parameters, including clogP, were calculated by using the free-access website https://www.molsoft.com/servers.html, accessed in 1 December 2021.

### 4.11. Statistical Analysis

Graph Pad Prism version 5.01 was used for the statistical analysis. Statistical analysis was performed via one-way ANOVA testing, followed by Bonferroni post-hoc testing. Data are represented as the mean ± SD; *p* values < 0.05 were considered statistically significant.

## Figures and Tables

**Table 1:** CC_50_, IC_50_, and SI for new synthetic compounds and standard NAIs against H5N1_wild_, H5N1_V116A_, H5N1_N295S_, and H1N1 viruses.

Compound Code	CC_50_ (µM)	IC_50_ (50% Inhibitory Concentration) (µM)				SI (Selectivity Index)			
		H5N1_wild_	H5N1_V116A_	H5N1_N295S_	H1N1	H5N1_wild_	H5N1_V116A_	H5N1_N295S_	H1N1
**7b**	1048	36.96	22.6	15.51	1.68	28.3	46.3	67.5	623.8
**8b**	788.4	8.633	16.2	19.6	1.7	91.3	48.6	40.2	463.7
**9b**	478.3	1.667	56.7	12.3	1.68	286.4	8.4	38.8	284.7
**7c**	764.5	1.62	11.26	37.4	1.65	472.5	67.9	20.4	463.9
**8c**	311.5	1.648	68.57	64.7	1.64	189	4.5	4.8	189.9
**9c**	302.0	2.246	1.65	55.09	1.64	134.4	183	5.4	184.1
**7a**	685.1	325	48.31	282.8	84.9	2.1	14.1	2.4	8
**8a**	503	26.3	923.8	22.81	1.61	19.1	0.54	22.0	312.4
**9a**	795.4	5.805	5.623	5.623	1.65	137	141.5	141.45	482
**zanamivir**	589.1	10.4	127.3	15.00	1.652	56.6	4.6	39.2	356.59
**oseltamivir**	580.8	10.1	309.2	25.8	1.650	57.5	1.878	22.5	352

Abbreviations: “CC_50_” half-maximal cytotoxic concentration; “IC_50_” half-maximal inhibitory concentration; “SI” Selectivity index = CC50/IC50.

**Table 2:** Liver and kidney function of mice after receiving the synthetic compounds.

Type of Analysis		ALT	AST	Creatinine	Urea
Compound Code	Control	6.5 ± 0.5	12.83 ± 1.81	0.30 ± 0.15	5.64 ± 2.17
**8b**	1st ^#^	10.66 ± 4.32	15 ± 3.41	0.17 ± 0.04	9.00 ± 3.81
	2nd	8.20 ± 2.62	15.77 ± 0.9944	0.13 ± 0.04	6.66 ± 1.54
	3rd	6.72 ± 1.47	16 ± 5.099	0.10 ± 0.02	7.32 ± 3.90
**9a**	1st	9.64 ± 3.64	20 ± 6.957	0.13 ± 0.04	7.32 ± 2.09
	2nd	10.64 ± 1.38 *	13.77 ± 1.298	0.13 ± 0.04	7.32 ± 0.48
		*p* * = 0.0085			
	3rd	8.67 ± 2.16	15.33 ± 0.9686	0.17 ± 0.04	4.64 ± 1.12
**9c**	1st	7.60 ± 1.56	11.93 ± 3.765	0.14 ± 0.05	7.90 ± 3.13
	2nd	7.80 ± 2.07	9.689 ± 2.922	0.14 ± 0.05	3.64 ± 1.49
			*p* * = 0.0395		
	3rd	5.54 ± 1.22	12.33 ± 3.169	0.13 ± 0.04	4.88 ± 2.13

Values are expressed as the mean ± SD and were analyzed by using one-way ANOVA with Tukey’s multiple comparisons test. * Significant at *p* < 0.05 when compared with control group. ^#^ The 1st Dose 20 mg/kg, 2nd Dose 40 mg/kg, 3rd Dose 80 mg/kg. Results were obtained by using a Robonik prietest ECO biochemistry analyzer. Alanine aminotransferase (ALT/GPT), Aspartate aminotransferase (AST/GOT), and serum creatinine were analyzed using the Kinetic method, but the blood urea was analyzed using a colorimetric method.

**Table 3:** Ligand efficiency of the best compounds against the NA of the H5N1 virus.

Compound	M.wt	Molecular Formula	NHA	cLogP	IC_50_ µM	pIC_50_	LE	LLE
**8b**	560.52	C_25_H_28_N_4_O_11_	40	1.73	6.863	8.16	0.28	6.43
**9b**	488.45	C_22_H_24_N_4_O_9_	35	1.67	1.679	8.77	0.34	7.10
**8c**	576.58	C_25_H_28_N_4_O_10_S	40	1.86	1.790	8.74	0.30	6.88
**9c**	504.51	C_22_H_24_N_4_O_8_S	35	1.80	2.280	8.64	0.33	6.84
**9a**	487.47	C_22_H_25_N_5_O_8_	35	1.24	2.75	8.56	0.34	7.32

M.wt: Molecular Weight, NHA: non-hydrogen atom, cLogP: lipophilicity, IC_50_: Inhibitory Concentration, pIC_50_: −log IC_50_ (indicates drug potency), LE: Ligand efficiency, LLE: ligand lipophilic efficiency.02D7.

## Data Availability

The data presented in this study are available within the article and Supplementary Materials.

## Supplementary Materials

The following supporting information can be downloaded at: https://www.mdpi.com/article/10.3390/ph15030351/s1, Figure S1: Titration of neuraminidase activity of H5N1wild, H5N1V116A, H5N1N295S, and H1N1 viruses; Figure S2: Safety of docked synthetic compounds (given as 3 doses), zanamivir, and oseltamivir compared with that of control (1X PBS). The safest compounds were **9c** and **9a**, which, like zanamivir, did not affect body weight. Compound **8b**, like oseltamivir, had a slight effect on body weight. Figure S3: Body weight loss of mice infected with different viruses that were given either synthetic compounds **8b**, **9c**, and **9a** 5 h post infection, oseltamivir or zanamivir as drug control, or no treatment (viral control [VC]). All groups were compared with an uninfected control group that was given 1X PBS instead of drugs or compounds. (A) Antiviral activity after infection with H5N1_wild_ (B) Antiviral activity after infection with H5N1_V116A_ (C) Antiviral activity after infection with H5N1_N295S_ (D) Efficacy and antiviral activity after infection with H1N1 virus; Figure S4: NMR spectra obtained by using a JEOL EX-500 spectrometer and (CDCl3) with TMS as internal standard.
