# Supplementary material for: In Vitro and In Vivo Antiviral Studies of New Heteroannulated 1,2,3-Triazole Glycosides Targeting the Neuraminidase of Influenza A Viruses
Source: Pharmaceuticals (Basel). 2022 Mar 14;15(3):351. doi: 10.3390/ph15030351 (PMC8950700; doi:10.3390/ph15030351)
Supplement: Supplementary file 1 [file pharmaceuticals-15-00351-s001.zip › pharmaceuticals-1551448-supplementary.pdf]

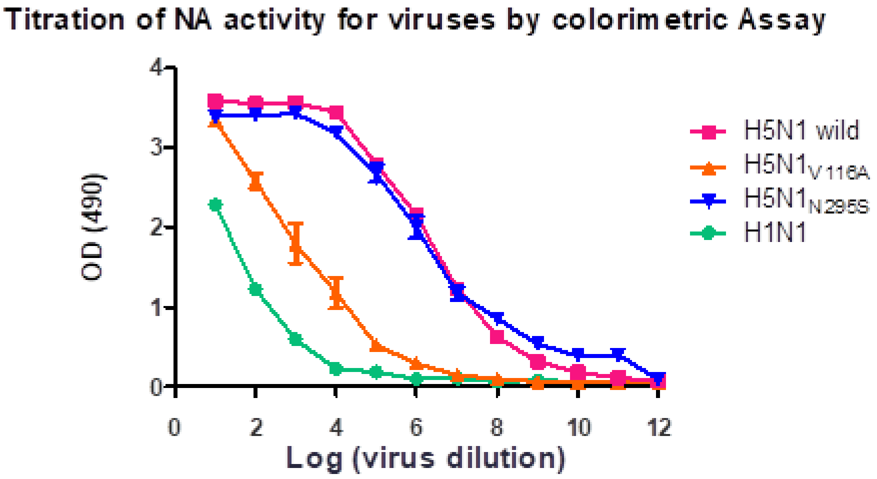

Figure S1. Titration of neuraminidase activity of H5N1wild, H5N1V116A, H5N1N295S, and H1N1 viruses.

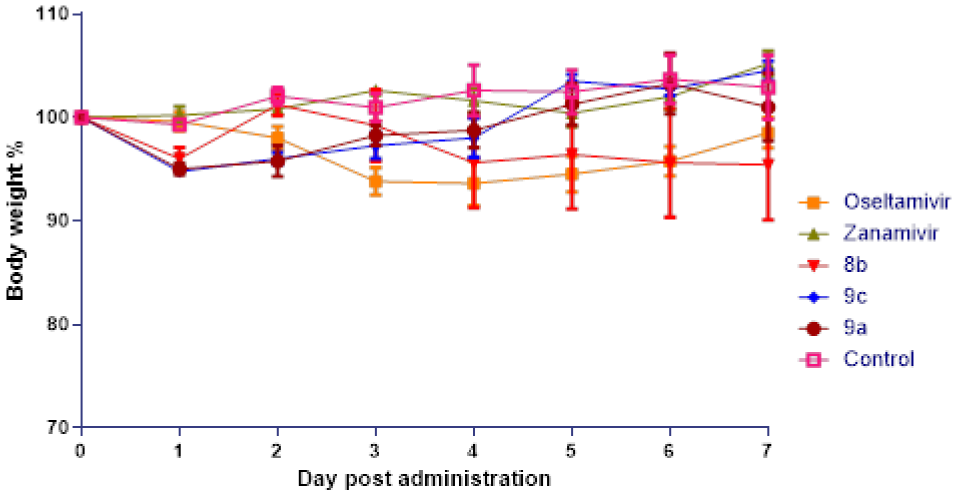

Figure S2. Safety of docked synthetic compounds (given as 3 doses), zanamivir, and oseltamivir compared with that of control (1X PBS). The safest compounds were **9c** and **9a**, which, like zanamivir, did not affect body weight. Compound **8b**, like oseltamivir, had a slight effect on body weight.

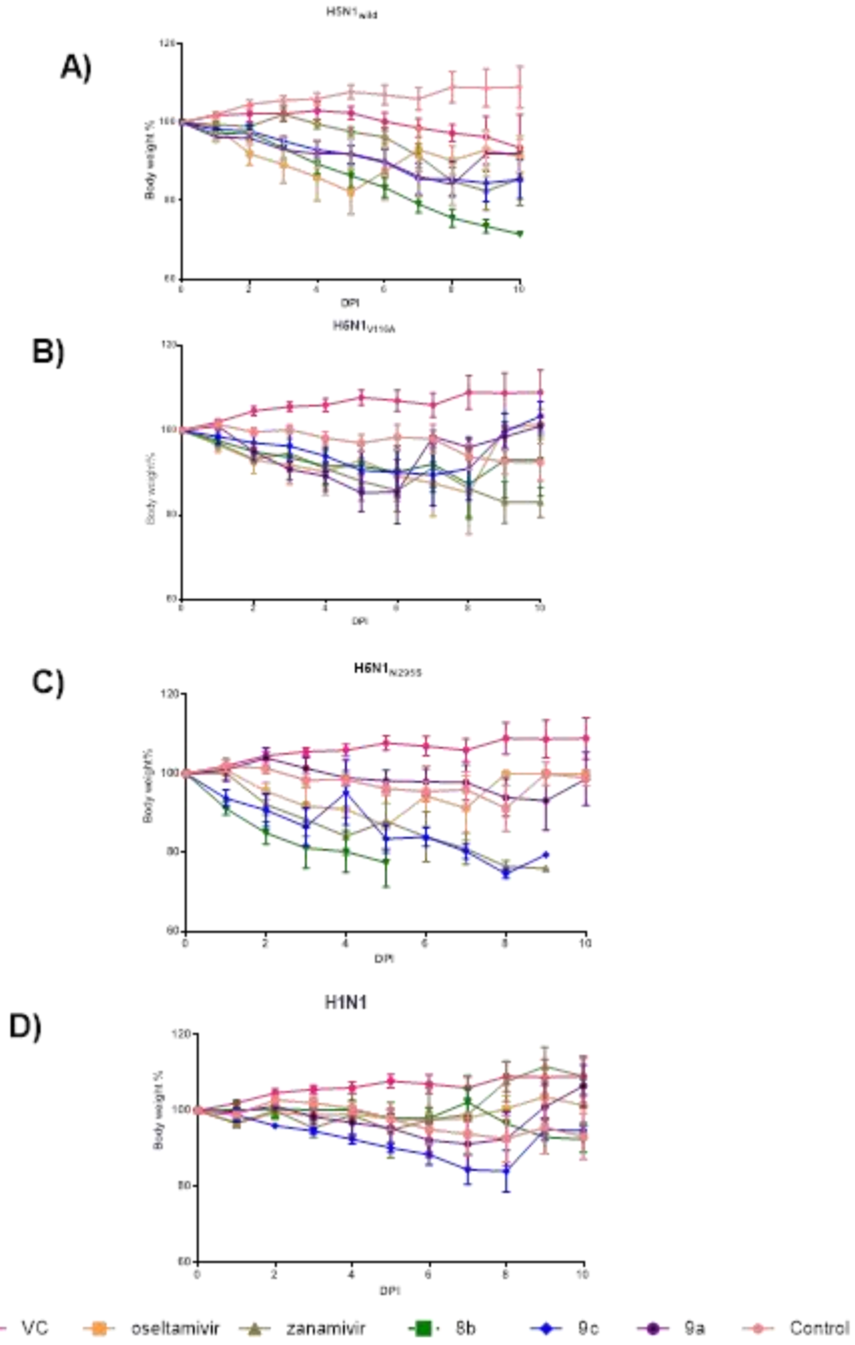

Figure S3. Body weight loss of mice infected with different viruses that were given either synthetic compounds **8b**, **9c**, and **9a** 5 h post infection, oseltamivir or zanamivir as drug control, or no treatment (viral control [VC]). All groups were compared with an uninfected control group that was given 1X PBS instead of drugs or compounds. (A) Antiviral activity after infection with H5N1$_{wild}$ (B) Antiviral activity after infection with H5N1$_{V116A}$ (C) Antiviral activity after infection with H5N1$_{N295S}$ (D) Efficacy and antiviral activity after infection with H1N1 virus.

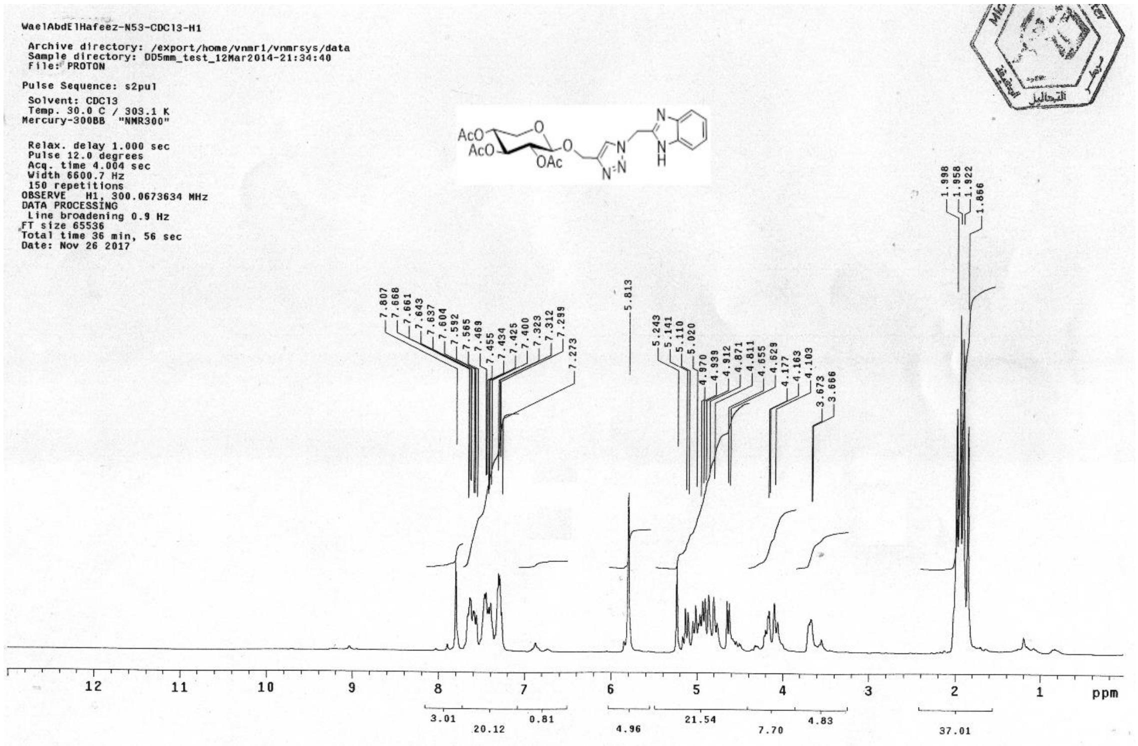
WaelAbdElHafeez-N53-CDCl3-H1
Archive directory: /export/home/vnmr1/vnmrsys/data
Sample directory: DD5mm_test_12Mar2014-21:34:40
File: PROTON
Pulse Sequence: s2pul
Solvent: CDCl3
Temp. 30.0 C / 303.1 K
Mercury-300BB "NMR300"
Relax. delay 1.000 sec
Pulse 12.0 degrees
Acq. time 4.004 sec
Width 6600.7 Hz
150 repetitions
OBSERVE H1, 300.0673634 MHz
DATA PROCESSING
Line broadening 0.9 Hz
FT size 65536
Total time 36 min, 56 sec
Date: Nov 26 2017
AcO
AcO
OAc
N
NH
7.807 7.668 7.661 7.643 7.637 7.604 7.592 7.565 7.469 7.455 7.434 7.425 7.400 7.323 7.312 7.299 7.273
5.813
5.243 5.141 5.110 5.020 4.970 4.939 4.912 4.871 4.811 4.655 4.629 4.177 4.163 4.103 3.673 3.666
1.998 1.958 1.922 1.866
12 11 10 9 8 7 6 5 4 3 2 1 ppm
3.01 20.12 0.81 4.96 21.54 7.70 4.83 37.01

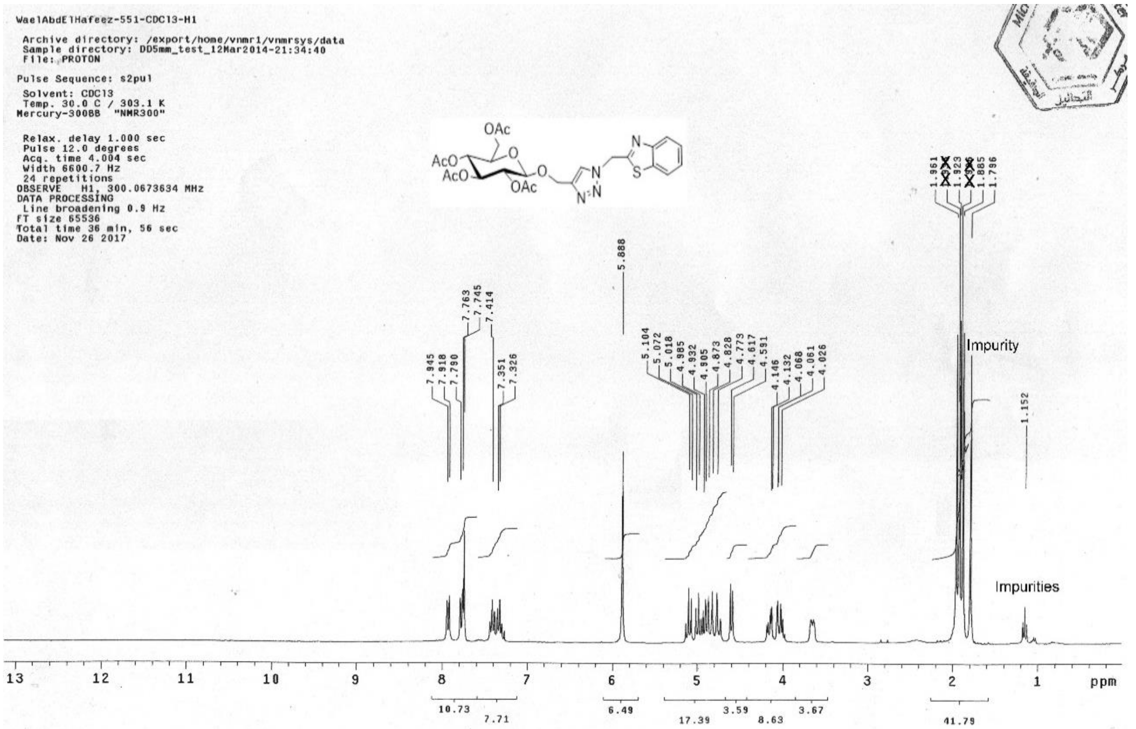
WaelAbdElHafeez-551-CDCl3-H1
Archive directory: /export/home/vnmr1/vnmrsys/data
Sample directory: DD5mm_test_12Mar2014-21:34:40
File: PROTON
Pulse Sequence: s2pul
Solvent: CDCl3
Temp. 30.0 C / 303.1 K
Mercury-300BB "NMR300"
Relax. delay 1.000 sec
Pulse 12.0 degrees
Acq. time 4.004 sec
Width 6600.7 Hz
24 repetitions
OBSERVE H1, 300.0673634 MHz
DATA PROCESSING
Line broadening 0.9 Hz
FT size 65536
Total time 36 min, 56 sec
Date: Nov 26 2017
OAc
AcO
AcO
OAc
N
S
7.945 7.918 7.780 7.763 7.745 7.414 7.351 7.326
5.888
5.104 5.072 5.018 4.985 4.932 4.905 4.873 4.828 4.773 4.617 4.591 4.146 4.132 4.068 4.061 4.026
1.961 1.923 1.885 1.796
Impurity
1.152
Impurities
13 12 11 10 9 8 7 6 5 4 3 2 1 ppm
10.73 7.71 6.49 17.39 3.59 8.63 3.67 41.79

TarekSaad-SS1-CDCL3-C13

Archive directory: /export/home/vnmr1/vnmrsys/data
Sample directory: DD5mm_test_12Mar2014-21:34:40
File: PROTON

Pulse Sequence: s2pul
Solvent: CDCl3
Temp. 30.0 C / 303.1 K
Mercury-300BB "NMR300"

Pulse 67.5 degrees
Acq. time 1.707 sec
Width 18761.7 Hz
328 repetitions
OBSERVE C13, 75.4520062 MHz
DECOUPLE H1, 300.0688576 MHz
Power 40 dB
continuously on
WALTZ-16 modulated
DATA PROCESSING
Line broadening 1.0 Hz
FT size 65536
Total time 3 hr, 6 min, 43 sec
Date: Oct 30 2018

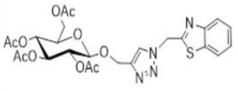

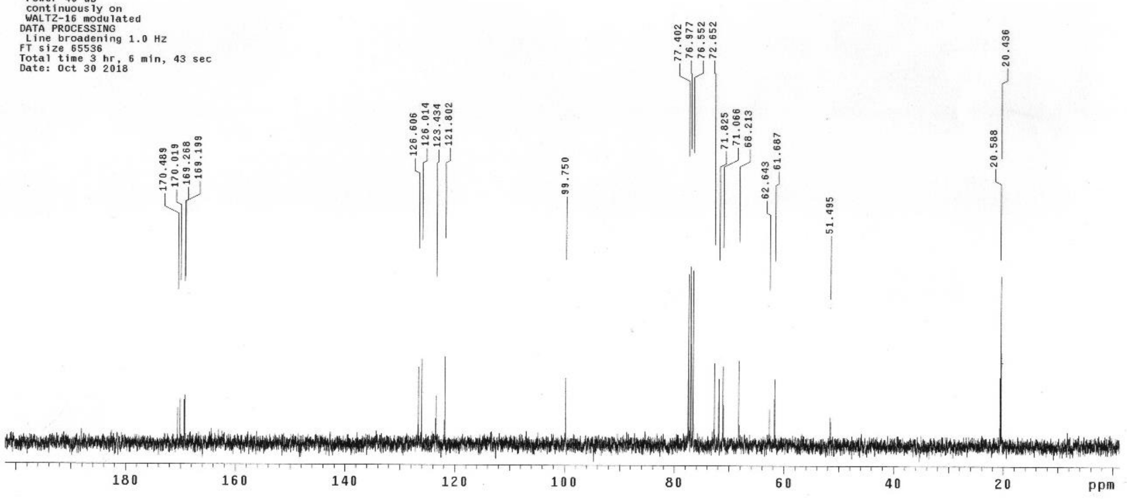

WaelAbdElHafeez-552-CDCl3-H1

Archive directory: /export/home/vnmr1/vnmrsys/data
Sample directory: DD5mm_test_12Mar2014-21:34:40
File: PROTON

Pulse Sequence: s2pul
Solvent: CDCl3
Temp. 30.0 C / 303.1 K
Mercury-300BB "NMR300"

Relax. delay 1.000 sec
Pulse 12.0 degrees
Acq. time 4.004 sec
Width 6600.7 Hz
57 repetitions
OBSERVE H1, 300.0673634 MHz
DATA PROCESSING
Line broadening 0.9 Hz
FT size 65536
Total time 36 min, 56 sec
Date: Nov 26 2017

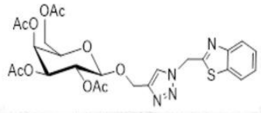

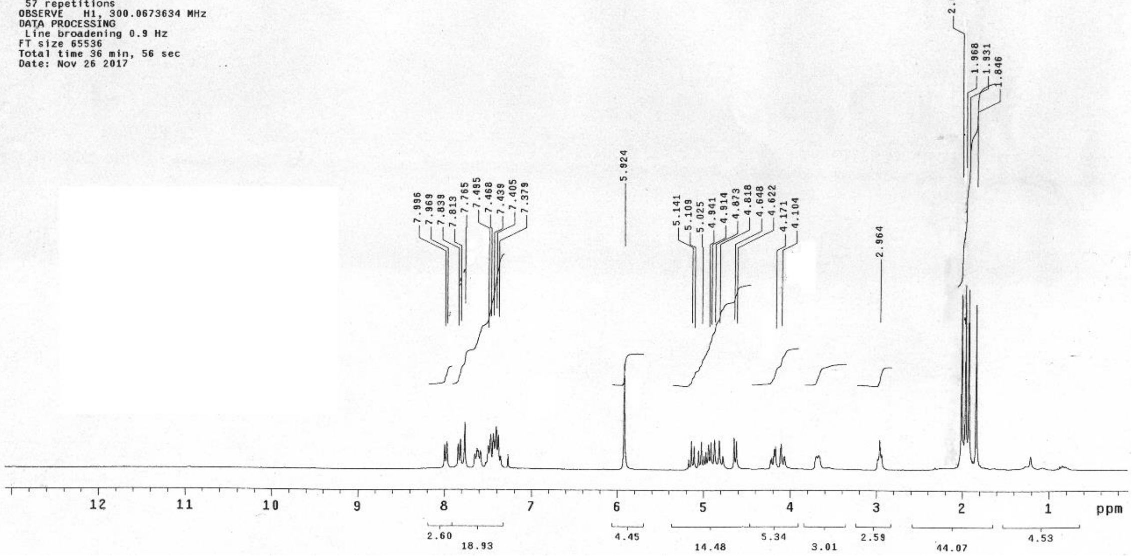

Tareq-SS2-CDCl3-13C

Archive directory: /export/home/vnmr1/vnmrsys/data
Sample directory: DD5mm_test_12Mar2014-21:34:40
File: PROTON

Pulse Sequence: s2pul

Solvent: CDCl3
Temp. 30.0 C / 303.1 K
Mercury-300BB "NMR300"

Pulse 67.5 degrees
Acq. time 1.707 sec
Width 18761.7 Hz
2032 repetitions
OBSERVE C13, 75.4520062 MHz
DECOUPLE H1, 300.0688576 MHz
Power 40 dB
continuously on
WALTZ-16 modulated
DATA PROCESSING
Line broadening 1.0 Hz
FT size 65536
Total time 3 hr, 6 min, 43 sec
Date: Oct 16 2018

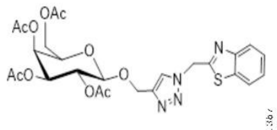

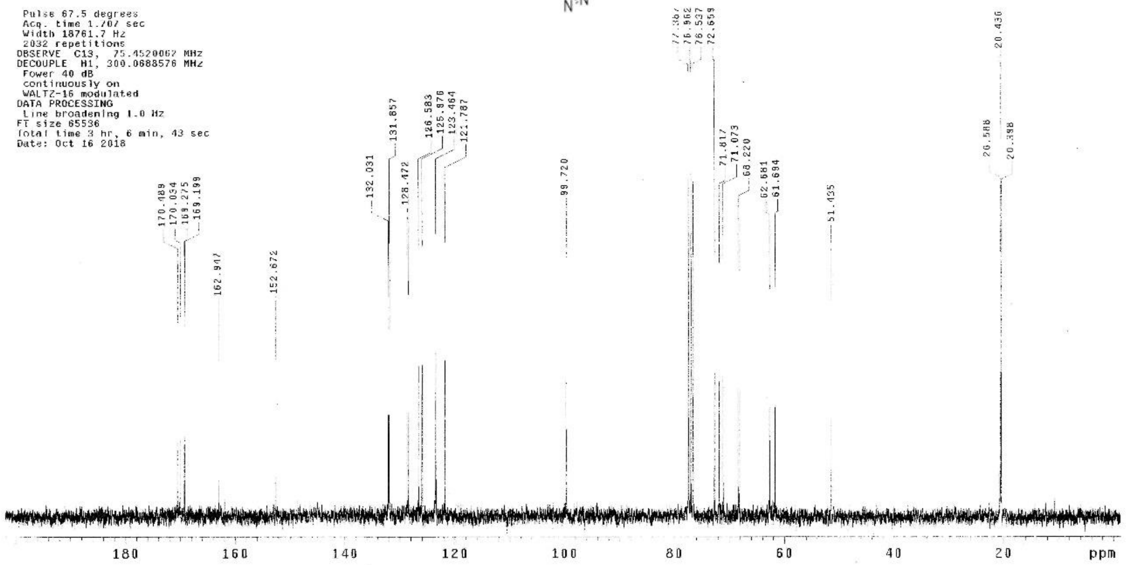

WaelAbdElHafeez-553-CDCl3-H1

Archive directory: /export/home/vnmr1/vnmrsys/data
Sample directory: DD5mm_test_12Mar2014-21:34:40
File: PROTON

Pulse Sequence: s2pul

Solvent: CDCl3
Temp. 30.0 C / 303.1 K
Mercury-300BB "NMR300"

Relax. delay 1.000 sec
Pulse 12.0 degrees
Acq. time 4.004 sec
Width 6600.7 Hz
46 repetitions
OBSERVE H1, 300.0673634 MHz
DATA PROCESSING
Line broadening 0.9 Hz
FT size 65536
Total time 36 min, 56 sec
Date: Nov 26 2017

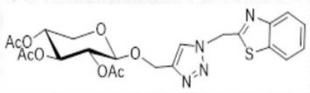

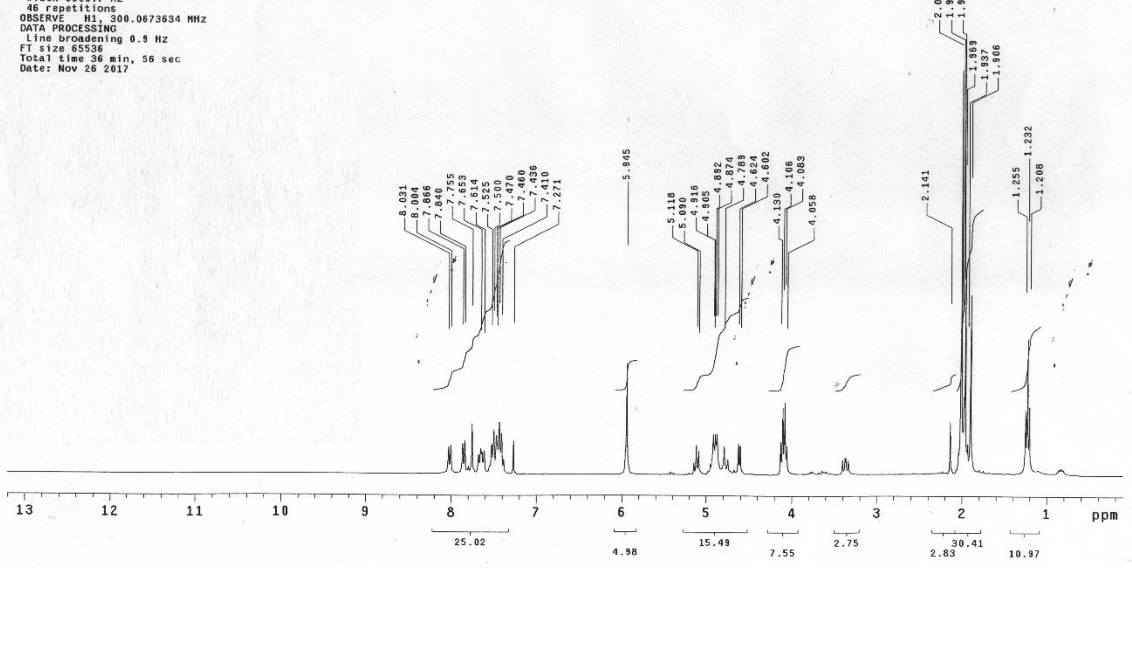

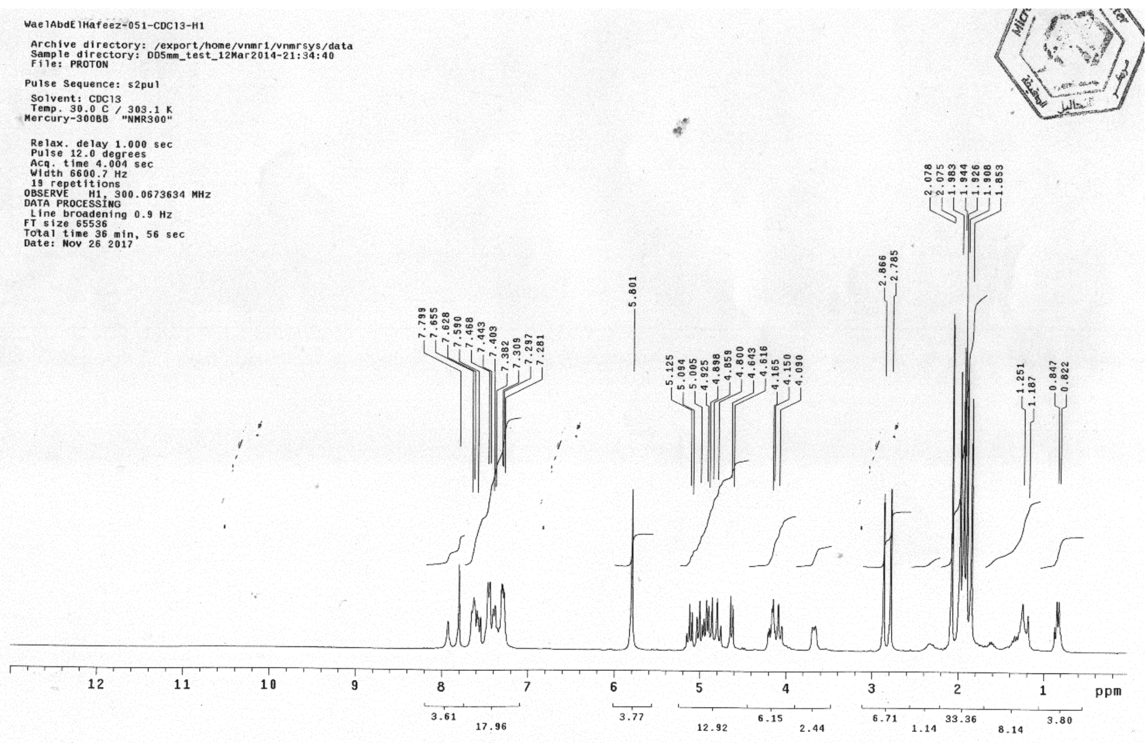
WaelAbdElHafeez-051-CDCl3-H1
Archive directory: /export/home/vnmr1/vnmrsys/data
Sample directory: DD5mm_test_12Mar2014-21:34:40
File: PROTON
Pulse Sequence: s2pul
Solvent: CDCl3
Temp. 30.0 C / 303.1 K
Mercury-300BB "NMR300"
Relax. delay 1.000 sec
Pulse 12.0 degrees
Acq. time 4.004 sec
Width 6600.7 Hz
19 repetitions
OBSERVE H1, 300.0673634 MHz
DATA PROCESSING
Line broadening 0.9 Hz
FT size 65536
Total time 36 min, 56 sec
Date: Nov 26 2017
7.799 7.655 7.628 7.590 7.468 7.443 7.403 7.382 7.309 7.297 7.281
5.801
5.125 5.094 5.005 4.925 4.898 4.859 4.800 4.643 4.616 4.165 4.150 4.090
2.866 2.785
2.078 2.075 1.983 1.944 1.926 1.908 1.853
1.251 1.187 0.847 0.822
12 11 10 9 8 7 6 5 4 3 2 1 ppm
3.61 17.96 3.77 12.92 6.15 2.44 6.71 1.14 33.36 8.14 3.80

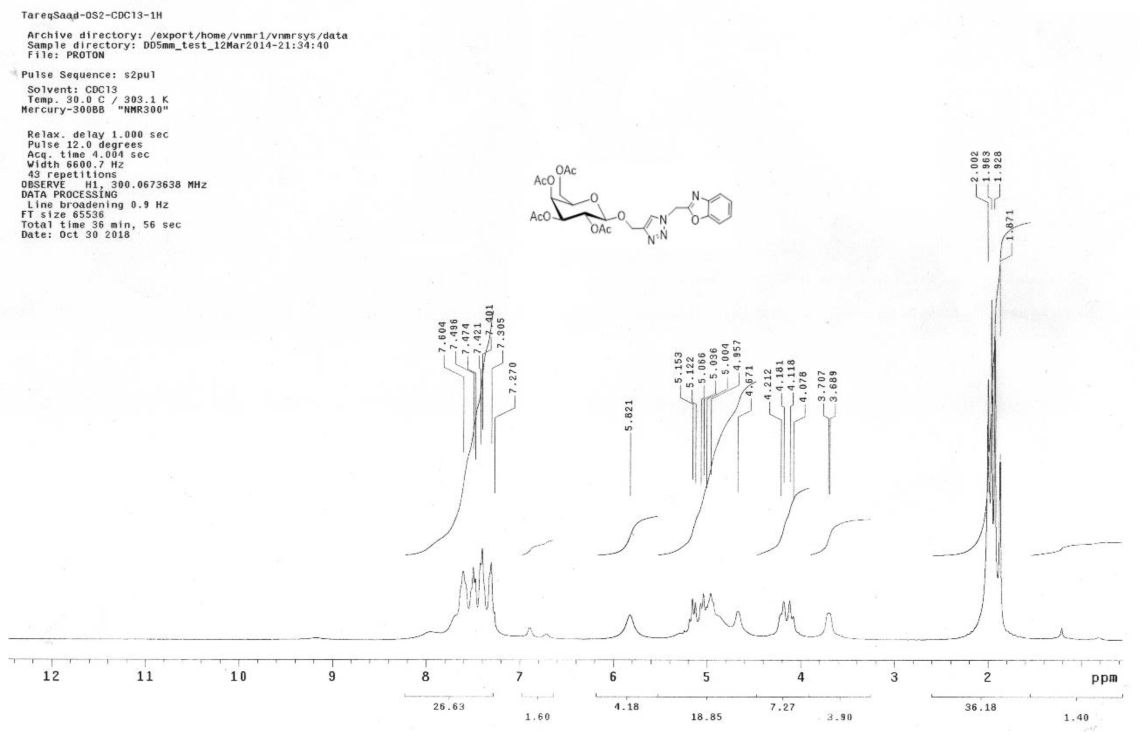
TareqSaad-OS2-CDCl3-1H
Archive directory: /export/home/vnmr1/vnmrsys/data
Sample directory: DD5mm_test_12Mar2014-21:34:40
File: PROTON
Pulse Sequence: s2pul
Solvent: CDCl3
Temp. 30.0 C / 303.1 K
Mercury-300BB "NMR300"
Relax. delay 1.000 sec
Pulse 12.0 degrees
Acq. time 4.004 sec
Width 6600.7 Hz
43 repetitions
OBSERVE H1, 300.0673638 MHz
DATA PROCESSING
Line broadening 0.9 Hz
FT size 65536
Total time 36 min, 56 sec
Date: Oct 30 2018
AcO
OAc
AcO
OAc
N
N
N
N
O
7.604 7.496 7.474 7.421 7.401 7.305 7.270
5.821
5.153 5.122 5.066 5.036 5.004 4.957 4.671 4.212 4.181 4.118 4.078 3.707 3.689
2.002 1.963 1.928 1.871
12 11 10 9 8 7 6 5 4 3 2 ppm
26.63 1.60 4.18 18.85 7.27 3.90 36.18 1.40

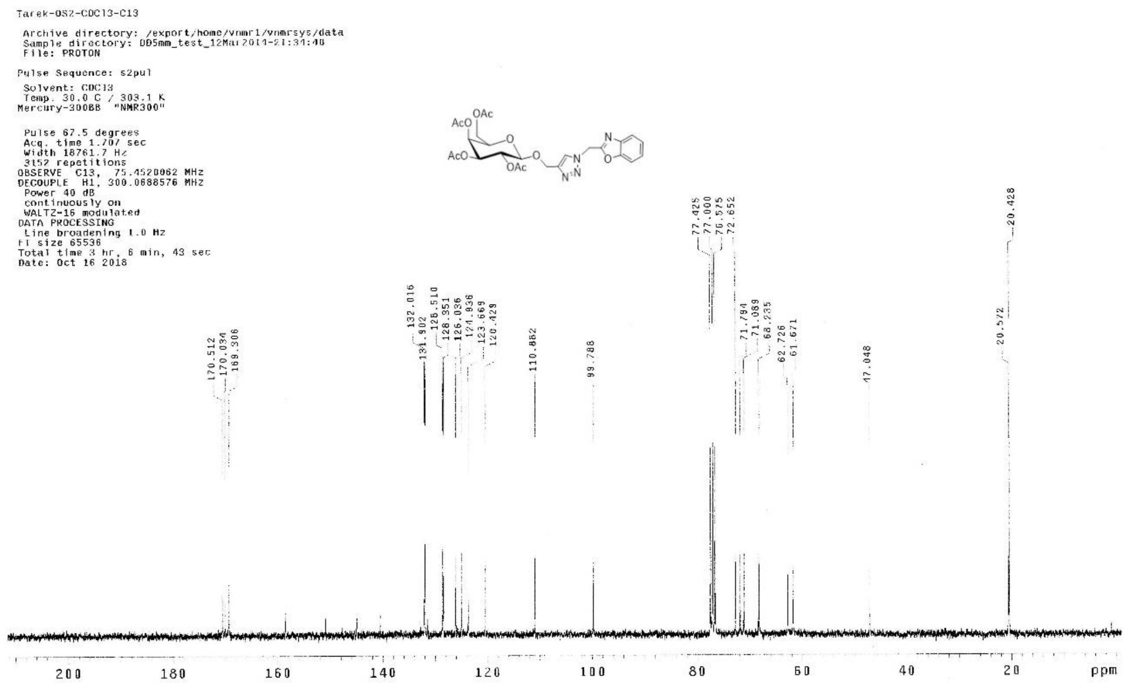

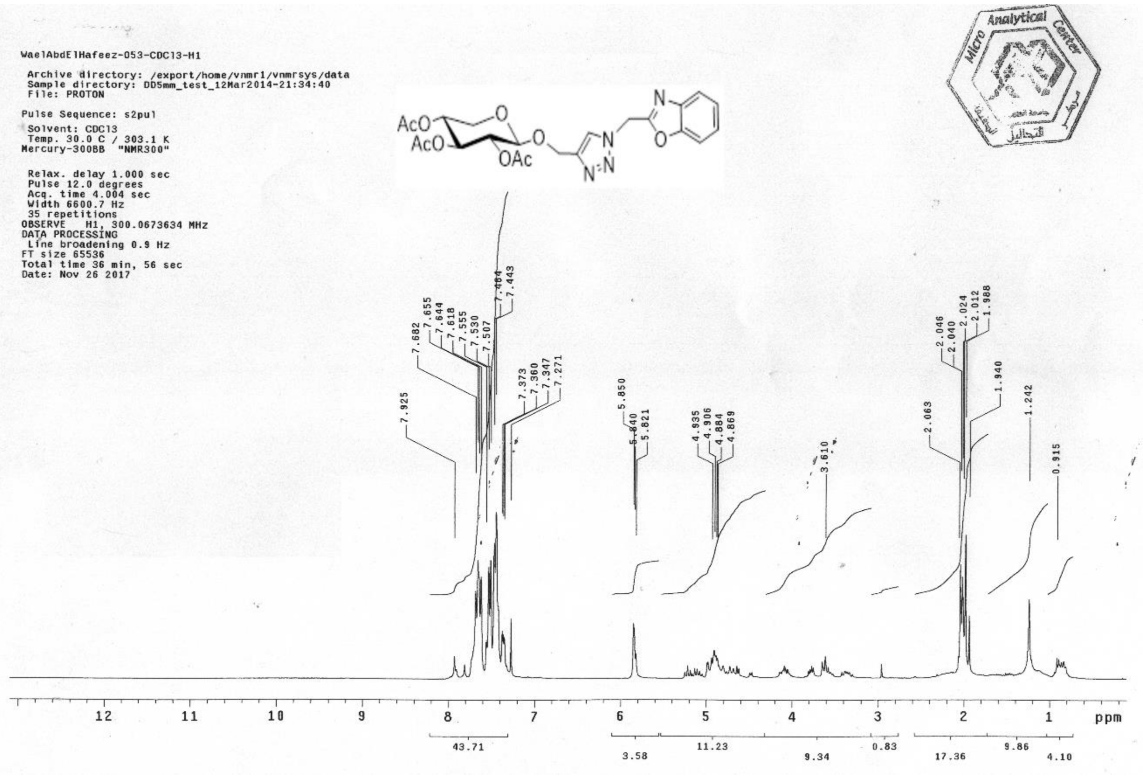

Figure S4 : NMR spectra obtained by using a JEOL EX-500 spectrometer and (CDCl3) with TMS as internal standard
